# Ultrasound mechanisms and their effect on solid synthesis and processing: a review

**DOI:** 10.1039/d4cs00148f

**Published:** 2024-10-23

**Authors:** Cedric Devos, Ariana Bampouli, Elena Brozzi, Georgios D. Stefanidis, Michiel Dusselier, Tom Van Gerven, Simon Kuhn

**Affiliations:** a KU Leuven, Department of Chemical Engineering Celestijnenlaan 200F 3001 Leuven Belgium tom.vangerven@kuleuven.be simon.kuhn@kuleuven.be; b Department of Chemical Engineering, Massachusetts Institute of Technology 77 Massachusetts Avenue, Cambridge MA 02139 USA; c School of Chemical Engineering, Department of Process Analysis and Plant Design, National Technical University of Athens Iroon Polytecneiou 9 Zografou 15780 Athens Greece; d Center for Sustainable Catalysis and Engineering (CSCE), KU Leuven 3001 Heverlee Belgium

## Abstract

Ultrasound proves to be an effective technique for intensifying a wide range of processes involving solids and, as such, is often used to improve control over both solids formation and post-treatment stages. The intensifying capabilities of ultrasonic processing are best interpreted in the context of the chemical, transport, and mechanical effects that occur during sonication. This review presents an overview of how ultrasound influences the processing and synthesis of solids across various material classes, contextualized within an ultrasound effect framework. By describing the mechanisms underlying the different effects of ultrasound on the solid synthesis and processing, this review aims to facilitate a deeper understanding of the current literature in the field and to promote more effective utilization of ultrasound technology in solid synthesis and processing.

## Abstract 2

Ultrasound intensifies a wide range of processes involving solids, enhancing control over their formation and post-treatment. This review summarizes key ultrasonic mechanisms and effects in solids' synthesis and processing.

## Graphical abstract

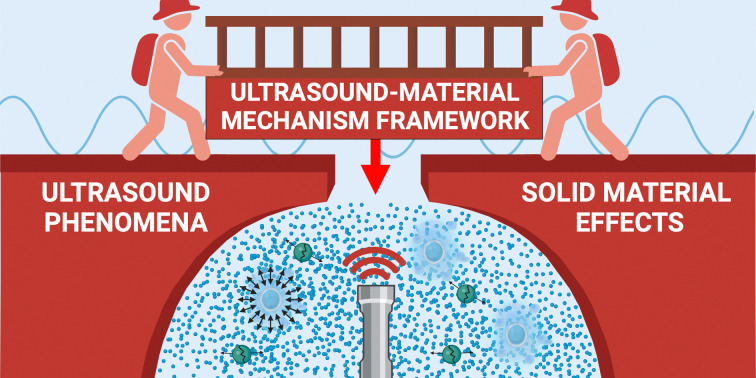

## 1 Introduction

The use of ultrasound (US) as an intensification technique applied during the synthesis and processing of solid materials can drastically improve the process efficiency, product quality, and selectivity. Its application may even render the addition of catalysts, conventional energy sources, or certain reagents superfluous.^1–4^ Moreover, ultrasound can be regarded as a sustainable technology, given its role in facilitating the electrification and low-impact manufacturing of solid materials.^5^

There is a growing body of literature and reviews illustrating the plethora of ultrasound applications for solids’ synthesis and processing. In 1967, Hem was the first to review the effect of ultrasonic vibrations on crystallization.^6^ Since then, several other reviews have discussed this topic (often called sonocrystallization) in more detail.^7–18^ Ruecroft *et al.* gave an overview of sonocrystallization for industrial applications.^7^ McCausland and Cains discussed the effect of power ultrasound on biomolecular crystallization.^8^ Deora *et al.* discussed the effect of ultrasound on crystallization in food processing.^18^ More recently, Jordens *et al.* summarized observations and theories in the field of sonocrystallization.^9^ Banakar *et al.* discussed ultrasound-assisted continuous crystallization in microreactors.^10^ Xiouras *et al.* reviewed the application of ultrasound in chiral crystallization processes.^15^ Moreover, several reviews in other fields than sonocrystallization were published throughout the years. Suslick *et al.* gave an overview of high intensity ultrasound on the processing of inorganic solids.^19^ Mckenzie *et al.* discussed the effects of cavitation in various media, focusing on sonochemistry of polymers with an emphasis on ultrasound-assisted radical polymerization.^20^ In a recent review by Kumar *et al.* the advantages of sonication on various polymerization mechanisms are outlined.^21^ Price *et al.* discussed polymer structure control with sonication.^22^ Basedow and Klaus gave an overview of the degradation of polymers in a sonicated solution.^23^ Other reviews have focused on the production of nanostructured materials using ultrasound.^24–26^ A tutorial review on the sonochemical synthesis of nanomaterials was published by Suslick's group^26^ and Cravotto *et al.* published a tutorial review on mechanochemical activation by ultrasound using case studies with crystals and polymers.^27^ Safarifard and Morsali reviewed ultrasound applications for the synthesis of metal–organic coordinated polymers.^28^ Skrabalak gave a perspective on the use of ultrasound for the synthesis and modification of carbon materials.^29^ Qiu and coworkers reviewed heterogeneous sonocatalysts’ characteristics.^30^ Shchukin *et al.* described the potential of solid surface functionalization using ultrasonication.^31^ Askari and colleagues described the effects of ultrasound on zeolite synthesis.^32^ Other reviews about zeolites touch briefly upon the use of ultrasonication during the synthesis step.^33–35^ In 2019, Vaitsis *et al.* examined the interaction between metal organic frameworks (MOFs) and ultrasound.^36^ Sonochemically prepared organic porous solids and their further applications in sonochemical processes were recently described by Koo and Kang.^37^ Athanassiadis *et al.* discussed ultrasound-matter interactions in smart materials.^38^

However, literature covering more than one material class, or discussions on the similarities and differences between different material classes are scarce and outdated. Two noteworthy exceptions from the past are the overviews given by Peters in 1996,^39^ and by Suslick and Price in 1999,^40^ both with a distinct emphasis on chemical ultrasonic effects (see Section 4.1).

The significance of sonoprocessing on solid materials continues to be relevant, as evidenced by the numerous recent publications on the subject.^20,21,41–44^ In the majority of cases, ultrasound is reported to produce better-than-benchmark results compared to conventional processing techniques. Ultrasound is also increasingly gaining traction as a viable technology in various industrial applications.^4^ In the pharmaceutical industry, it has been notably utilized to enhance productivity and to improve product quality during particle formation processes.^4,27,45^ Similarly, ultrasound is now considered as an economically viable processing technology in several food processes in which solids are handled.^46^ Moreover, disintegration of sludge particles was successfully scaled-up with ultrasonic technology.^47^ The use of ultrasound has also become established for specific niche applications in materials science and nanotechnology: it is, for example, considered the primary technique for dispersing (*i.e.*, properly distributing solids in liquids) nanofillers.^48,49^ Despite this, there continues to be a critical gap in establishing direct relationships between ultrasonic phenomena and the reported benefits in solid–liquid systems. A deeper understanding of this connection would, on the one hand, enable scaling up of processes that are now limited to the laboratory scale;^47^ on the other hand expand and further establish the application of ultrasound to several other industrial processes dealing with suspensions and slurries where it has already been proven effective in academia, such as sand cleaning from contaminated oil,^50^ and acceleration of meat curing processes^51^ (see also Section 7). In this review, an overview of the effects of ultrasound during different stages of solids’ processing is given by linking the ultrasonic phenomena and mechanisms to their effects on solid materials. Such a point of view distinguishes this review from others in the field, as it deliberately deviates from the more narrow focus found in existing reviews. To do this, an engineering perspective is adopted, wherein experimental observations in solid materials are classified in a generalized framework of ultrasonic mechanisms. This integrative approach allows for a more nuanced understanding of the role of ultrasound in solids’ processing through isolation and disentanglement of different phenomena often occurring simultaneously. While ultrasonic processing might be perceived as a niche technique with impressive yet unpredictable results, this review demonstrates that understanding core mechanisms provides key insights into a variety of systems and processes. The effects of ultrasound on the synthesis and modification of biological solids (*e.g.*, ref. 21, 40 and 52–56) are not included here. Moreover, despite this restriction, similar to Peters in 1996,^39^ we still acknowledge the vast expanse of information in this field and recognize the difficulty of encompassing every detail. In light of this, our aim is not exhaustive coverage, but rather a panoramic overview of effects and mechanisms that occur across various material classes to offer insights for a more targeted use of ultrasonic technology for the synthesis and processing of solid materials.

This review is structured as follows: Section 2 covers the historical perspective and current research trends in the field of ultrasound for solids’ processing. Section 3 gives insights into the general phenomena occurring in solution when ultrasound is applied. Section 4 presents the framework in terms of the different ultrasonic (subdivided into chemical, mechanical, and transport) effects and mechanisms that occur in the presence of solids. While this section covers general ultrasound-liquid–solid mechanisms, Section 5 discusses experimental observations in terms of these effects and mechanisms across different material classes. This work concludes with a summary and a future outlook.

## 2 Ultrasound and solids in research

### 2.1 A historical perspective of ultrasound in solids processing

Following substantial technological advancements in ultrasonic technology during the First World War, ultrasound soon found application in solids’ processing: one of its earliest recorded applications occurred in 1927 when ultrasound was utilized in a crystallization process,^57^ with little success. Depolymerization by ultrasonication followed soon after, with initial reports dating back to 1933.^58^ With advances in piezoelectric technology, the use of ultrasound applications also began to increase substantially, especially after the Second World War.^13^ For instance, the effects of ultrasonication on enhancing the kinetics of crystallization processes and reducing particle size were already well studied in the 1960s and 1970s.^6,59–61^ The term ‘sonochemistry’ was coined in 1953 to describe chemical effects originating from ultrasound,^62^ but it was not until around 1980 that ultrasound was systematically applied in heterogeneous reactions to enhance kinetics and modify surface morphology.^40,63^ Since the 1990s, ultrasound has also been applied to influence more intricate solid synthesis and post-treatment processes, for example for porous structures,^64^ and for carbon nanotubes.^65^

Since its first use almost a century ago, ultrasound has manifested itself as an established technology for enhancing solid synthesis and processing, with a plethora of effects that can be exploited. Beyond increasing process kinetics (during the synthesis or assembly stage), sometimes by several orders of magnitude, ultrasound is probably best known for its ability to reduce particle size and fine-tune solid properties in the post-treatment stage. Most reports predominantly focus on applications in the low frequency regime (see Section 3.1 for details on the different regimes and their consequences), although in the last decade also the benefits of the high frequency regime have been increasingly explored (*e.g.*, ref. 66 and 67). The effects of both regimes have also been exploited simultaneously by alternating between two different frequencies.^68^

### 2.2 Current state of the art

The maturity of ultrasonic processing of solid materials varies significantly among different material classes. For instance, sonocrystallization has attracted significant interest over the years, maintaining a steady level of publications per year.^69^ Similarly, ultrasound for polymers processing has been steadily rising in the past 20 years.^69^ Sonoprocessing of metal powders observed a boost at the end of the previous century.^69^ In contrast, the application of ultrasonication during the processing of porous structures has only emerged in recent years, with a steep increase in the number of publications observed in the past five years.^69^ This demonstrates that ultrasound technology continues to reach new fields. For instance, the potential of ultrasonication during the synthesis of covalent organic frameworks (COFs) is still to be fully explored, with only a few examples currently in the literature.^70–75^ Thus, the sections of this review concerning ultrasonic processing in porous structures focus predominantly on zeolites, which currently dominate the ultrasound-porous materials field.

## 3 The fundamentals of ultrasound phenomena

**Fig. 1:**
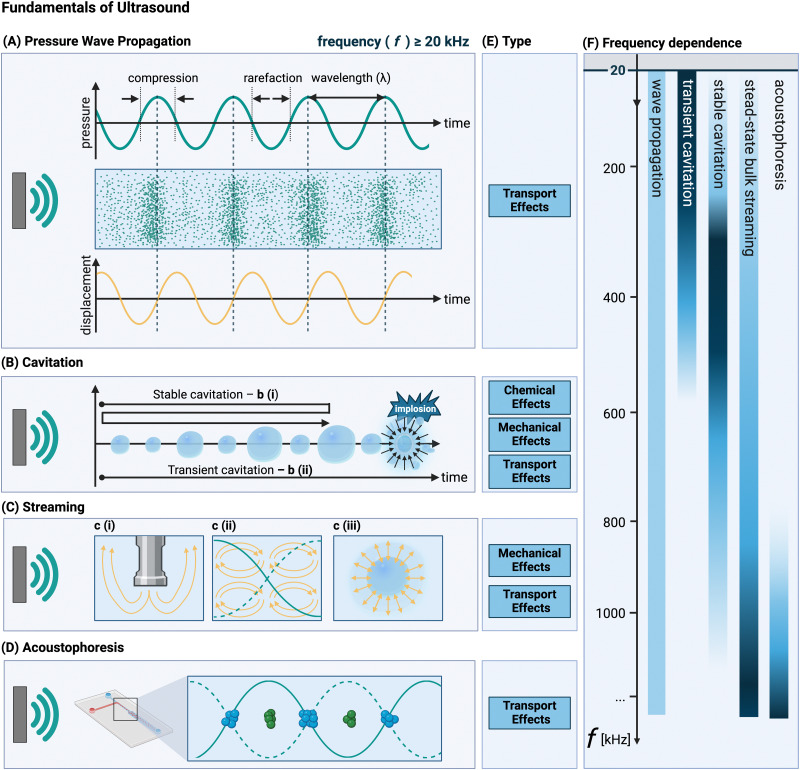
Schematic overview of the three ultrasonic phenomena that occur in solution during ultrasonic processing: (A) ultrasonic wave propagation: a (longitudinal) acoustic wave (with wavelength *λ*, and frequency (*f* ≥ 20 kHz) propagating through a solution, with the molecules of that medium undergoing alternating cycles of compression and rarefaction in the wave direction; (B) cavitation: the formation and growth of cavitation bubbles (stable, b (i) or transient, b (ii)); (C) acoustic streaming: fluid flow as a result of viscous attenuation. c (i) Eckart streaming, c (ii) Rayleigh streaming, and c (iii) microstreaming associated with the oscillatory movement of a cavitation bubble; (D) acoustophoresis: focussing of solid particles at the pressure nodes of a standing acoustic wave (here shown in a microfluidic chip); (E) the effect types associated with the ultrasonic phenomena; (F) typical frequencies at which the different ultrasonic phenomena are most pronounced, with darker colors indicating greater importance and lighter colors indicating lesser importance. If no color is shown, the effect is considered negligible at that frequency. Created with https://Biorender.com.

Ultrasound refers to all longitudinal acoustic waves above the human hearing frequency (about 20 kHz).^46^ Acoustic waves are generated from the oscillatory motion of a transducer's surface adjacent to a solution, which is induced by an electrical energy input.^38^ To exploit ultrasound for solids’ synthesis and processing, the acoustic energy, propagated by acoustic waves, must be transferred into the system through a liquid phase^46^ and converted into the desired energy form – whether it be physical, mechanical, or chemical. This transferring and conversion occurs through three core phenomena: ultrasound wave propagation, cavitation, and acoustic streaming (see Fig. 1). Cavitation is the most utilized phenomenon in solids formation and processing, with acoustic streaming following with lesser importance. In some applications involving solids, the acoustic radiation force is exploited, leading to a phenomenon known as acoustophoresis.

These different phenomena that occur in solution interact with solid materials in different ways, which can be classified into different categories: chemical, transport, and mechanical effects. Chemical effects refer to those effects in which a chemical reaction takes place. Transport effects group effects that affect mass and heat transfer. Mechanical effects originate from mechanical energy being released in the solution generating mechanical forces.

### 3.1 General considerations

A distinction is made between high-frequency ultrasound (HFUS, with frequencies above 1 MHz) and low-frequency ultrasound (LFUS, with frequencies below 1 MHz, with the 20–100 kHz range being the most used one).^76,77^ This is an important categorization (although the transition region is quite wide^68,77^) as the ultrasonic effects that arise from the interactions between the acoustic waves and the liquid medium through which it travels differ between LFUS and HFUS. LFUS applications in a liquid solution usually operate at power inputs above the transient cavitation threshold (see Section 3.3), to generate acoustic cavitation microbubbles. In the 20 to 100 kHz frequency range, the number of bubbles is low, but they tend to have a larger size at resonance. As a result, in this range the effects on transport properties are most pronounced.^4^ Although longer collapse times are observed at lower frequencies,^4^ the implosion of larger-sized bubbles tends to lead to the strongest mechanical forces.^42^ As the ultrasonic frequency increases, the presence of a higher number of antinodes in the pressure waves leads to the formation of an increased number of smaller bubbles.^78^ This results in more noticeable chemical effects, particularly in the transient region, for frequencies up to 500 kHz or 1 MHz.^4^ This reduction in the oscillation period eventually leads to a strongly dampened – if any – collapse.^4^ Moreover, due to technical limitations, HFUS is usually operated at lower powers, which, combined with a larger power loss towards molecular motion,^20,47^ prevents the solution from reaching the acoustic pressure cavitation threshold (see Section 3.3).^77^ As a consequence, acoustic streaming is the dominant effect (and no chemical effects are observed). Research on the effect of ultrasound on solids’ processing has been primarily focused on LFUS and on the effects of cavitation associated with it. The application of HFUS is mainly described in the literature in case of solid handling to prevent/counteract deposition and clogging, without any specific reference to its transport or mechanical effects on the system.^79^

Ultrasound can be applied directly (*e.g.*, with a probe in a solution) or indirectly (*i.e.*, with a solid surface separating transducer and solution).^35^ Ultrasonic generators or transducers (*e.g.*, probe, piezo-element, ultrasonic bath), reactor design, material, and roughness all have a major impact on the ultrasonic field topology and the final product properties. Discussions concerning various transducers and ultrasonic reactor types suitable for solid synthesis and processing applications are considered out-of-scope, but can be found in a number of reviews.^4,77,79–82^

### 3.2 Acoustic wave propagation

Ultrasound waves are oscillatory mechanical vibrations that propagate through a medium, thereby inducing small disturbances in the molecules’ positions,^83,84^ see Fig. 1(A). Acoustic waves are best described by parameters related to their propagation through the medium. These include pressure, particle velocity and displacement, and density fluctuation. Typically, acoustic systems are operated under harmonic driving conditions (*i.e.*, in periodic conditions), and therefore undergo sinusoidal oscillations at a single frequency (*f*, see Fig. 1(A)). Consequently, the aforementioned quantities oscillate at this driving frequency over time, resulting in the formation of periodic waves in space. These waves are characterised by a wavelength (*λ*) which is defined as speed of sound in the medium divided by the frequency.‡‡This relationship is valid for waves of small amplitude, the presence of significant nonlinearities or large amplitudes may lead to the emergence of additional harmonics.^38^ ^38^ The intensity of ultrasound through the liquid medium is inversely proportional to the medium's impedance (defined as the density multiplied by the speed of sound), which represents to what extent the liquid is in tension when a wave passes through. This is a good measure for the probability of cavitation bubble collapse (see Section 3.3).^83,85^

Acoustic waves can efficiently propagate even through complex media. When solids are present in the solution, the sound waves undergo reflection, transmission, or refraction at the fluid-solid interface. In such cases, the distribution of ultrasonic energy within the heterogeneous solution depends on its capacity to absorb and propagate that energy efficiently. The intricacies of wave propagation within the solids is out of scope for this review, but has been covered in literature.^86^

The strength of the ultrasonic field can be quantified and reported through various operational parameters. The pressure amplitude (also called acoustic amplitude^87^), measured in pressure units, denotes the magnitude of the acoustic wave (typical values range from 0.1 bar, for a weak ultrasonic field, up to several bars).^87^ Sometimes the nominal electrical input voltage of the transducer and amplifier is reported. The power (*P*), quantified in Watt, signifies the total energy transferred and is proportional to the square of the amplitude.^88^ The nominal net power (usually reported as just the power, with typical values ranging from anywhere between 1 and 1000 W^47^) delivered to the transducer accounts for the difference between input amplifier and reflected power. For comparing different transducers, as is sometimes done for ultrasonic horns, the power radiating from the tip (see further) is best reported in terms of the power intensity (*i.e.*, the power passing through a unit area normal to the sound propagation direction – expressed in units of Watt per square meter – with typical values for moderate intensity ranging from 1 to 20 W cm^−2^).^87^ Describing ultrasonic effects in a system benefits from reporting the power density, *i.e.*, power divided by total volume (with standard values ranging from 0.01 to 2 W mL^−1^, though some studies show significant deviations from these values), especially when considering scale up of ultrasonic systems. Calorimetric power, derived from measuring the bulk temperature change due to ultrasonication (see further) over a specific time, is arguably the most accurate for comparing studies across varying frequencies, as highlighted in the literature (*e.g.*, ref. 89–91), as it is a measure for the effective applied power.

### 3.3 Acoustic cavitation

Acoustic cavitation is the most important phenomenon exploited during solid synthesis and processing using ultrasound. It encompasses the formation, growth and sometimes rapid collapse of gaseous microbubbles in a liquid as a result of sonication.^3,4,20,76,81^Fig. 1(B) illustrates graphically how tensile forces during the rarefaction (or negative pressure) cycles in the acoustic wave cause growth of the cavitation bubble. Cavitation bubbles are classified as either stable or transient,^4,80,92^ schematically presented in Fig. 1(B). Stable cavitation consists of oscillating bubbles that remain stable and do not implode for a long time.^42,92,93^ During these oscillation cycles the dissolved gases in the liquid migrate through the cavitation bubbles, cushioning the implosion upon collapse.^80^ On the contrary, transient cavitation bubbles survive only for a few acoustic cycles,^92,94^ which does not provide sufficient time for the dissolved gas to attenuate the bubble collapse.^94^ In the case of transient cavitation, once the bubble has reached a critical threshold size (determined by the ultrasonic frequency), it collapses violently.^42^ The gas within the bubble undergoes rapid quasi-adiabatic compression,^42^ which causes a localized region in the solution with high temperatures (>5000 K^95,96^) and pressures (>1000 atm^96^), *i.e.*, transport phenomena (see Section 4.3). Bubble collapse is often approximated as adiabatic as the total collapse time is very short (in the ns-range).^97^ The conditions within and surrounding the imploding bubble are so extreme that they can induce molecular dissociation,^42^*i.e.*, chemical phenomena (see Section 4.1).

**Fig. 2:**
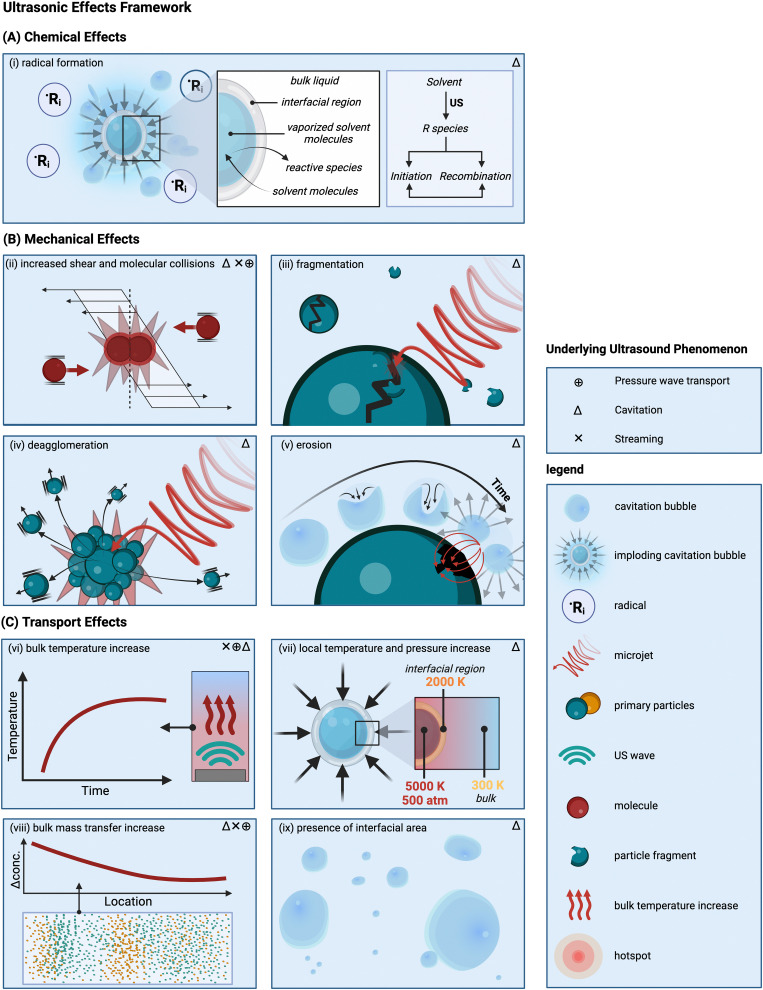
Overview of ultrasonic effects and the potential mechanisms affecting solids’ processing. (A) Chemical effects: (i) the formation of radical species due to the sonolysis of solvent molecules. (B) Mechanical effects: (ii) shear-induced molecular collision, (iii) fragmentation of solid particles due to shockwaves associated with symmetric bubble implosions, (iv) deagglomeration of solid agglomerates as a result of shockwaves associated with symmetric bubble implosions; (v) erosion or surface modifications of the surface of a solid particle as a result of microjets associated with asymmetric bubble implosions. In the case of layered materials exfoliation can occur. (C) Transport mechanisms: (vi) bulk temperature increase, (vii) appearance of local transient temperature and pressure hotspots, (viii) local concentration gradients as a result of bubble collapse and improved bulk mass transfer, and (ix) presence of additional interfacial area. The top right corner of every effect shows the underlying ultrasonic phenomenon in decreasing order of importance. Created with https://Biorender.com.

Cavitation implosion is a high-energy localized event,^40^ leading to the generation of mechanical phenomena and stresses, associated with liquid jets (for asymmetric collapse due to the presence of a nearby (solid) surface larger than the imploding bubble radius, which is around 150–200 μm for sonication at 20 kHz (as shown in Fig. 2B(v))),^98^ and shockwaves (for symmetric or spherical collapse, as shown in Fig. 2A(i)) in the region surrounding the bubble (*i.e.*, 1 to 25 μm in diameter).^20,99^ The shockwave front can reach pressure values of 100 to 1000 MPa for a couple of ns.^99^ Shockwave velocities of up to 2000 m s^−1^ have been recorded.^42,100^ The resulting jets, with diameters one-tenth of the original bubble size,^99^ can reach liquid velocities of up to 100 m s^−1^, with impact pressures of 200 MPa over a 0.05 to 0.5 ns time interval,^42^ thereby exceeding typical material threshold velocity values above which erosion occurs (see Section 5).^42^

Mathematical descriptions of bubble nucleation and dynamics can provide insights into the required ultrasonic parameters (*e.g.*, frequency and power) for cavitation, and can be applied on different scales, ranging from the microscale of the nucleation of a single bubble to the macroscale of an entire system. The probability of nucleation of a cavitation bubble in a homogeneous, pure liquid (which rarely occurs) is described by the classical nucleation theory,^101,102^ however the most common analytical method to estimate the parameters for transient cavitation onset in a more realistic heterogeneous environment is *via* calculation of the Blake threshold pressure. It describes the minimum pressure required for spontaneous unlimited bubble growth in quasi-static pressure conditions.^46,103,104^ Bubble dynamics equations usually make use of the Blake threshold to model the dynamic expansion and subsequent bubble collapse upon reaching the critical radius:^103^ the most used equations for this purpose are the Rayleigh–Plesset equation and the Keller–Miksis equation. The former is accurate for most of the cavitation bubble growth and collapse duration, but disregards the compressibility of the liquid. The latter, an extension of the Rayleigh–Plesset equation, accounts for liquid compressibility.^103,105^ The true dynamics of a cavitation bubble are dependent on several parameters, such as fluid flow, liquid viscosity and its tensile strength, or the presence of dissolved gases.^102,104,106^ For instance, an increase in viscosity will make overcoming the cavitation threshold more difficult,^80,105^ and will modify the bubble collapse dynamics within the system.^105–108^ As a result, direct correlations among different systems are challenging to establish, but some trends can still be discussed. At the system scale, numerical simulations are needed to quantify cavitation behaviour in flow, which account for bubble dynamics and motion, acoustic pressure distribution, fluid dynamics, mass transfer and sometimes reaction kinetics.^103,104,109^ The numerical approaches are categorized according to how the liquid and gas phases are considered.^109^ The single Euler phase model only looks indirectly at the bubbles by modelling the changes in the liquid phase. Euler–Euler models, instead, look at the gaseous and the liquid phases as continua. Lagrangian tracking methods treat cavitation bubbles as discrete entities and focus on their motion. A combination of these models is the Euler–Lagrange method, which treats the liquid phase as a continuum and the bubbles as particles.^109^ Usually the choice of the most suitable model relies on the spatial and temporal scales that need to be described:^109^ to date, there is no ‘one size fits all’ model capable of providing a general description of cavitation phenomena occurring in the liquid phase. Moreover, due to the complexity of the system, most available models rely on single bubble dynamics, neglecting effects such as clustering, bubble–bubble coalescence, and rectified diffusion.^110^ Single bubble models can be validated experimentally, becoming a valuable tool for ultrasonic performance optimization.

Understanding ultrasonic effects often, at least in the LFUS regime, hinges on accurately characterizing cavitation activity and its distribution throughout the system. Sutkar and Gogate have given an overview of experimental and numerical approaches to achieve this.^81^ Also Ashokkumar has discussed cavitation characterization,^92^ and more recently, Meroni *et al.*^4^ provided a guide for selecting appropriate techniques for monitoring various sonication effects. Experimentally, the cavitational activity can be measured through pressure and temperature recordings, using techniques like calorimetry, thermal visualization, and hydrophone measurements. Quantification of the effects accompanying the collapse of cavitation bubbles can be performed *via* dosimetry (*e.g.*, iodine, Fricke, terephthalic acid), sonochemiluminescence of luminol and electrochemical methods, particle image velocimetry and aluminum foil erosion.^4,81^

### 3.4 Acoustic streaming

In addition to the regular oscillatory motion induced by the ultrasound wave propagation, also acoustic streaming can occur if the wave propagates through a viscous fluid.^111^ Acoustic streaming is defined as any fluid flow generated from the attenuation of an acoustic wave.^112^ Several types of streaming may arise, as shown in Fig. 1(C), typically divided based on the length scale and geometry of the flow.^112^ Two types of bulk streaming can occur: If the acoustic wavelength is considerably smaller than the characteristic length scale of the reactor (*i.e.*, in large reactors), the streaming is called Eckart streaming (Fig. 1c(i)). If the acoustic wavelength is larger than the characteristic length scale of the reactor^113^ (which is valid for microfluidic reactors) boundary-layer driven streaming can occur.^112^ By generating a standing wave parallel to the surface, this streaming phenomenon can be exploited in microreactors to generate rotating vortices (called Schlichting and Rayleigh streaming (Fig. 1c(ii)).^112^ A third type of streaming, cavitation microstreaming (Fig. 1c(iii)), arises from the movement of stable oscillating cavitation bubbles, generating flow through viscous dissipation in their boundary layers.^112^ As Wiklund *et al.* point out, this is not to be confused with jets created upon cavitation implosion (which are not classified as acoustic streaming, but as acoustic flow).^112^

In general, acoustic streaming depends upon the applied power and frequency of ultrasound, and typically ranges from the order of μm s^−1^ (*i.e.*, slow streaming) to a few cm s^−1^ (*i.e.*, fast streaming).^76,112^ The resulting flow patterns are associated with an increase in the local heat and mass transfer (which in turn affects the bulk transfer) and disturbance of the pressure field formed due to the scattering of the acoustic waves.^76,81^ Investigations into the acoustic streaming field, encompassing the velocity magnitude, the vector direction, and flow patterns have been conducted using both numerical simulations and experimental methods for water and increased viscosity solutions.^67,106,114^

### 3.5 Acoustophoresis

If an acoustic standing wave is generated within a microreactor in the HFUS regime, acoustic radiation forces can push μm-sized particles towards the pressure nodes, a phenomenon called acoustophoresis (or acoustophoretic motion).^115^ By adjusting the operating parameters, the particles’ position can be tuned.^116^ The source of these acoustic radiation forces is the scattering of the acoustic waves on the particles.^115^ This effect is commonly leveraged for particle handling in microfluidic devices, primarily to prevent clogging (*e.g.*, for calcium carbonate and barium sulfate precipitation^117^), size-based particle and cell sorting (*e.g.*, for a suspension of glycine crystals),^116^ and cell aggregation (*e.g.* red blood cells focusing without damage).^118^ Given that the primary application of acoustophoresis is managing solid transport or trapping material at specific locations, its effects on the solids themselves during post-treatment are limited. Therefore, acoustophoresis is not discussed in this review. The interested reader is referred to a series of tutorial reviews.^115,118–127^

## 4 A mechanism framework for understanding ultrasonic effects

In this section a framework is proposed to identify and classify the different ultrasonic effects that occur during solid synthesis and processing. Moreover, it relates these effects with the underlying ultrasonic phenomena. This categorization of mechanisms and effects as proposed here does not imply that the different mechanisms cannot occur simultaneously or that the associated effects are not connected to each other. In fact, the boundaries between these effects often “remain blurred”.^79^ It is also important to recognize that the framework includes mechanisms that are theoretically plausible but have not (yet) been observed experimentally.

### 4.1 Mechanism related to the chemical effects

This category groups all effects that are caused by the presence of ultrasound-generated radicals.^3,128^ The generation of free radicals can be attributed to the implosion of millions of (transient) cavitation bubbles.^81^ Although radicals form in solution, in some cases before any solids are present, their generation is important as they initiate key chemical reactions.

The most prevalent theory explains the formation of radicals through the appearance of localized transient high-pressure zones and temperature hotspots during the cavitation bubbles implosion,^81,95,129^ which can cause the rupture of vaporized solvent molecules into radicals.^130^ This theory considers three possible reaction zones: the interior of a cavitation bubble, the bubble–liquid interface, and the bulk of the liquid.^131^ The zone where the chemical reaction occurs depends on the physical and chemical properties of the molecules involved. Specifically, volatile molecules traverse the bubble–liquid interface, as shown in Fig. 2(A), encountering extreme temperature and pressure conditions that result in the breakage of their chemical bonds within the bubble cavity.^3,24^ Here, organic molecules will produce reactive species *via* homolytic bond breakage. Subsequently, these newly formed chemical species can either return to the bulk liquid and undergo further reactions, or recombine and react with gases before engaging in other reactions.^131^ This depends on the bubbles’ lifetime compared to the radicals’ lifetime, which is typically longer at low frequencies (*e.g.*, around 10^−5^ s at 20 kHz) and shorter at higher frequencies (*e.g.*, around 4 × 10^−7^ s at 500 kHz).^3^ At medium-range frequencies (from 200 to 800 kHz)^132^ the majority of the reactions, such as free-radical and pyrolysis-like reactions, occur at the interface, where the efficiency of bond cleavage is usually the highest.^131,132^ In the bulk liquid, however, no primary (or gas-phase) radical reactions occur, but the diffusion of radicals or oxidizing species to the bulk can cause some secondary (or solution-phase) activity.^24^

In the case of reactions happening inside the bubble, for aqueous solutions, cavitation causes water to dissociate into hydrogen atoms and hydroxyl free radicals (*i.e.*, sonolysis).^3,24,85^ As previously mentioned, these highly reactive species can either recombine or initiate reactions within the collapsing bubble, at the bubble–liquid interface or in the bulk.^24,85^ The sonolysis of water entails both primary and secondary reactions, and – if present – can also involve secondary gases such as O_2_, as illustrated in eqn (1) (with US = ultrasound).^3,21,24^1
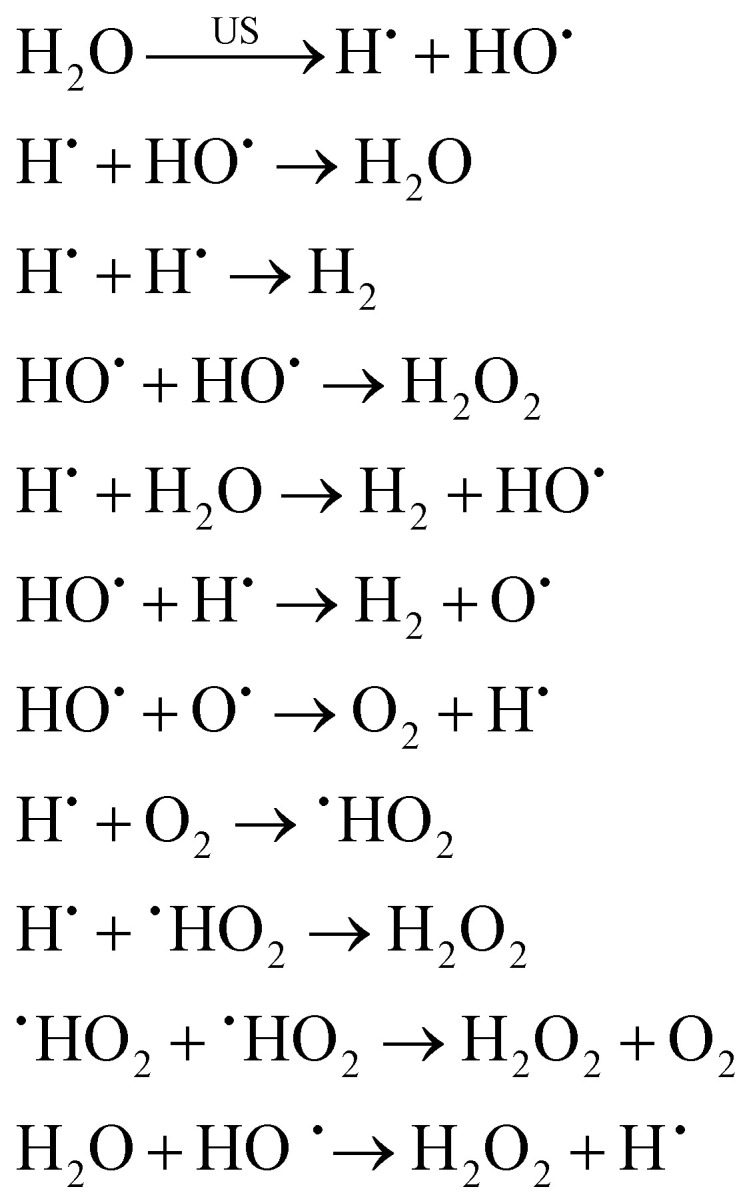


Volatile organic compounds in aqueous environment can react with these oxidizing species, forming different radicals, recombination products, or newly formed products.^132^ Sonolysis can also occur in nonaqueous solvents, although it typically occurs with lower intensity, and follows the same trends as the vapor pressure of the solvents. Nevertheless, other fundamental parameters – such as surface tension and viscosity, as well as dissolved gas concentration – should be taken into account for a more accurate evaluation.^3,85,132,133^ Riesz *et al.* have reported in detail on the chemical effects in both aqueous and nonaqueous solvents,^85^ with examples including products originating from carbon tetrachloride, chloroform and alkanes based radicals. In a later study,^134^ radical types were identified for numerous additional solvents. A summary of a selection of organic solvents and their radical products is presented in Table 1.

**Table 1:** List of selected organic solvents and their possible radical products, with R, R_1_ and R_2_ representing alkyl functional groups. Adapted with permission from Misik and Riesz.^134^ Copyright 1994 American Chemical Society

Solvent	Typical radical products
*n*-alcohols	˙CH_2_ R, ˙CH(OH)R, ˙CHR_1_R_2_
*n*-alkanes	˙CH_2_ R, ˙H, ˙CHR_1_R_2_
Dimethylformamide	˙CH_3_, ˙CH_2_ N(CH_3_)COH, ˙C(O)N(CH_3_)_2_
Cyclohexane	˙Cycloxehyl ring
Dioxane	˙Dioxane
Toluene	˙CH_2_-phenyl, ˙CH_3_
Chloroform	˙CHCl_2_
Carbon tetrachloride	˙CCl_3_
Dimethylacetamide	˙CH_3_, ˙C(O)N(CH_3_)_2_, ˙CH_2_ N(CH_3_)COCH_3_
Methylformamide	˙CH_2_ N(H)COH, ˙N(CH_3_)COH, ˙C(OH)N(H)CH_3_

The radical and carbene intermediates presented in Table 1 can recombine resulting in more stable products. For the case of chloroform, for instance, various final products can be obtained, as shown in eqn (2):^85^2
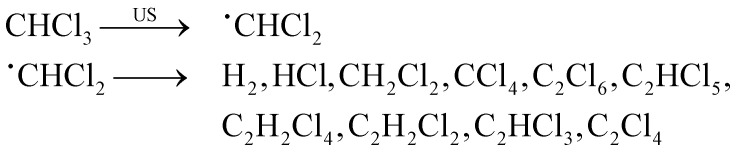


Reactions at the gas–liquid interface occur *via* indirect mechanisms.^132^ In this category, apolar volatile compounds undergo pyrolysis, whereas amphiphilic compounds are first hydrolized before reacting with the products of water sonolysis. Eventually, the compounds that tend to react in the bulk of the solution are usually either non-volatile or strongly hydrophilic.^132^ A detailed mechanistic insight is considered out of scope for this review, but can be found in the literature.^3,24,85,131–133^

**Fig. 3:**
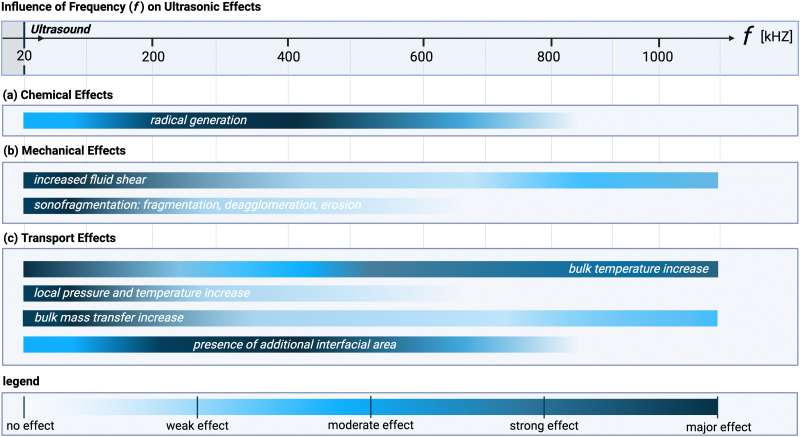
Acoustic wave frequency dependence of the different ultrasonic effects within a specific system at constant temperature and pressure: (a) chemical, (b) mechanical, and (c) transport effects. The color gradient represents the intensity of the effect: dark colors indicate that the effect is most pronounced at the corresponding frequency, while light colors indicate a weaker importance. This figure assumes a constant net power across all frequencies and that the system is optimized to exploit the dominant ultrasonic phenomenon at each specific frequency (*e.g.*, through reactor design, medium characteristics, *etc.*). Note that the bars indicate the extent to which each effect is dependent on frequency, not the relative importance of each effect, which is highly system-dependent. Created with https://Biorender.com.

Radical generation does not follow the same frequency trend as cavitation intensity, which can be seen by comparing Fig. 1(F) with Fig. 3. At 20 kHz, where transient cavitation is most pronounced, the relatively long (about 10^−5^ s) bubble collapse time gives sufficient time to radicals to undergo recombination reactions. An increase in the ultrasonic frequency, leads on one hand, to a lower number of radicals per bubble (as the bubble size decreases and less heat is generated),^78^ while on the other hand, it causes the bubble collapse time to become shorter than the lifetime of most radicals.^3^ Moreover, the number of bubbles increases, which leads to an overall increase of radicals concentration in the system until a maximum is achieved. By increasing the ultrasound frequency further, the rarefaction cycle duration decreases up to a point where there is insufficient time for implosion of the cavitation bubble. At frequencies above 1–2 MHz (HFUS) cavitation is completely suppressed, and acoustic streaming becomes the dominant mechanism.^77^

Recently some research has suggested that chemical effects may also occur in the absence of cavitation as a result of ultrasonic wave propagation compressing the liquid to form a solid-like transient state, which develops charges that can enhance electron mobility.^44,135^

The appearance of radicals generated from cavitation implosion is exploited in the field of sonochemistry.^80,95,129^ While sonochemistry can occur in both homogeneous and heterogeneous systems, for the scope of this review only the latter will be described.^136^ Heterogeneous sonochemistry can be further subdivided into two categories: reactions involving non-organometallic compounds and reactions involving metals, with the latter being the most studied ones.^3,136^ Ultrasound can accelerate the reaction rate by promoting surface depassivation, see Section 5.2.3, followed by facilitation of the transfer of an electron from the metal to the organic acceptor molecule.^3^ The underlying mechanisms are still subject of debate.^132,135,137,138^ One hypothesis suggests that the acoustic field has the ability of decreasing the band gap for electron transfer in the metal. Another one proposes that the shockwaves caused by bubble collapse excerpt mechanical stresses on the solids, where energy is stored in the form of lattice defects. Its release (*via* heat, emission of active species, bond breakage) is responsible for reaction rate enhancement.^138,139^

The collapse of cavitation bubbles is not always sufficient to generate a considerable number of radicals to synthesize or degrade the desired solid.^140^ One viable approach to augment the chemical effects within the solution is to introduce a solid phase capable of amplifying cavitation phenomena.^3,140–142^ These solids act as “catalysts” for the chemical reaction taking place in the solution. This phenomenon is defined as heterogeneous sonocatalysis and it is based on the interaction between ultrasound and surface chemistry (see Section 4.3).^30^ As a whole, sonocatalysis has been explored in the direction of sustainable chemistry as an alternative to the use of toxic solvents to synthesize organometallic reagents, to reduce overall energy consumption by speeding up reactions when electron transfer from metal surface is the rate-limiting step, and to improve product yield and selectivity.^3^

### 4.2 Mechanisms related to the mechanical effects

Fig. 2(B) illustrates important mechanical effects and their underlying mechanisms observed during ultrasonic processing of solids. Mechanical effects are all the effects originating from mechanical forces in the solution or stresses induced in the solids. These effects can be attributed to the generation of shockwaves and microjets from cavitation collapse, and to a lesser extent to microstreaming near oscillating cavitation bubbles and steady-state acoustic streaming. Which mechanical effects of ultrasound can occur depends on the structural solid scales present in solution.

At the molecular level (*e.g.*, during solid synthesis), the primary mechanical effect is an increase in molecular collisions. This can be attributed to an increased fluid shear within the liquid. As shown in Fig. 3, fluid shear is highest for low frequencies, *i.e.*, where cavitation intensity peaks due to secondary fluid flow arising from bubble collapse. In the transient region, there is little to no increase in fluid shear, until it rises again, driven by the acoustic streaming phenomenon.^4,9,94,112^ At larger scales, the mechanical effects are referred to as sonofragmentation and they are directly related to the intensity of cavitation bubble collapse. Consequently, these effects are most pronounced in the LFUS regime (see Fig. 1(F) and 3.^4^ Sonofragmentation encompasses both particle breakage and erosion.^143^ However, they stem from two different and well-defined mechanisms.^144^ Cavitation erosion is defined as the surface damage caused by the impingement of solvent microjets onto a solid surface following asymmetric bubble collapse.^4,107^ Particle breakage mechanisms, on the other hand, originate from shockwaves, a result of symmetric collapse of cavitation bubbles,^130^ and are further divided into two different categories depending on the type of bonds affected. Fragmentation refers to breakage of intraparticle bonds within a primary particle.^145^ Deagglomeration, instead, refers to the breakage of the interparticle bonds between particles aggregated in agglomerates into smaller agglomerates or removal of primary particles from the agglomerated cluster itself.^145^ Both fragmentation and deagglomeration, despite being different phenomena, cause an increase in the surface-to-volume ratio of the solid material. For example, particle fragmentation typically results from short intense stress peaks on a particle, while erosion, like abrasion, occurs due to the cyclic application of lower-intensity stresses on particle surfaces.^144^ Mechanical effects depend on intrinsic material properties; fragmentation, for example, is determined by factors like tensile strength, density, friability, malleability, melting point,^94,98^ toughness (*i.e.*, the ability of a material to absorb energy prior to fragmentation), brittleness, the Vickers hardness, the Youngs modulus,^9,146^*etc.* The elasticity of a solid material can predict its ability to deform, prior to fragmentation. Polymers, for instance, are known to deform, but not break under limited sonication. Apart from the mechanical properties of the single material units, also the solids size, size distribution, and shape may play a role. When particles are too large they will not accelerate sufficiently to cause considerable effects, and when they are too small, they are less likely to collide.^11^ Despite this size dependency, in specific cases, particle fragmentation can continue to occur into the submicron scale.^20,143^

### 4.3 Mechanisms related to the transport effects

All the effects related to the formation or presence of gradients within the system, such as concentration, pressure, and temperature changes, are grouped under the transport effects category. The common transport effects with their originating mechanisms are schematically summarized in Fig. 2(C). The most noticeable among them is an initial temperature increase of the solution^147^ when irradiated, which eventually levels off to a constant steady-state value. This effect can be attributed to nonlinear acoustic irradiation, viscous attenuation of the acoustic wave, and thermal conduction in presence of cavitation.^38,148^ Systematic trends for bulk temperature increases in ultrasonic systems are complex to evaluate, as they are usually estimated *via* calorimetric measurements, which are highly system dependent. Additionally, within systems, operation at resonance frequency or at sub/superharmonics will also affect the thermal dissipation. The highest bulk temperature increase usually occurs in the LFUS regime, followed by the HFUS regime, due to strong viscous attenuation. However, in the HFUS regime the temperature rise is often limited as lower ultrasonic powers are used compared to operation in LFUS regime. In addition to this bulk effect, the implosion of cavitation bubbles results in local transient temperature and pressure hotspots in the solution layers near the implosion. This effect is closely related to the intensity of bubble cavitation collapse, and, therefore, follows the same frequency trend (see Fig. 1(F) and 3).^149^

It is also hypothesized that cavitation implosion shockwaves can introduce local concentration gradients in the solution by segregating molecules based on their relative density.^9^ The segregation hypothesis proposes that cavitation implosion shockwaves create local concentration gradients in solution by segregating molecules such as large molecules or nanoparticles, from the host liquid based on their relative density.^150–154^ Denser solute molecules are separated from less dense solvent molecules, with high-density solute clusters remaining close to the collapsing bubble.^150–154^ The extent to which concentration gradients form then depends on the density of the solid and the solvent in which it is dispersed.

Furthermore, steady fluid movement induced by ultrasound (*i.e.*, cavitation streaming, steady-state streaming, and wave propagation) improve bulk mass transfer if concentration gradients are present in solution, even within heterogeneous systems. The frequency dependency of effects related to bulk mass transfer is complicated to assess. In the LFUS regime, the violent cavitation implosions enhance mass transfer considerably. In the HFUS regime, in geometrically optimized systems, bulk mass transfer improves through steady-state bulk streaming. However, a current limitation is that transducers operating at higher frequencies often lack sufficient power, resulting in milder effects.

Lastly, the presence of thousands of cavitation bubbles in the solution means that there is a significant amount of additional interfacial gas–liquid area present in the reactor, which can affect the solids’ processing, as seen in crystallization with gas bubble injection (*e.g.*, ref. 155–158). This is usually not considered to have a (significant) impact on the solids’ synthesis and processing due to the dominance of other ultrasonic effects. The ultrasonic frequency significantly influences both the size and number of bubbles formed (see Section 3.1). As the frequency increases towards the transition region, the number of cavitation bubbles increases while their size decreases, leading to a greater gas–liquid interfacial area within the system. This trend continues until a threshold in the HFUS regime is reached, beyond which bubble formation ceases and cavitation is suppressed.^4^

Additionally, the mere presence of solids in solution can influence the system by helping trigger bubble formation, thanks to a shift from a homogeneous to a heterogeneous nucleation mechanism. This induces more shockwaves and microjets following asymmetric collapse, enhancing cavitation activity and its consequences (also mechanical and chemical).^3,30,31^

### 4.4 Dependence of effects on ultrasonic parameters

The intensity of the effects presented in this section depends on the reactor configuration and on the selected ultrasonic parameters, such as, frequency, power density, and sonication time. Increasing sonication time, while keeping power and frequency constant, enhances the observed effects up to a plateau, beyond which further increases yield minimal additional impact.^159^ An increase in applied power (for constant sonication time and frequency) usually leads to an increase in the observed effects, although in some cases, the use of high power leads to the formation of excessive cavitation bubbles, which dampen the signal (a phenomenon known as acoustic shielding).^160^

The relationship between the different effects and the acoustic frequency is more nuanced, as shown in Fig. 3. Certain effects exhibit convex behavior, being strongest at a central frequency and tapering off at higher and lower frequencies. In contrast, effects with concave behavior are weakest at the central frequency and become more pronounced at the extremes. The key distinction is that convex effects result from a single ultrasonic phenomenon (*e.g.*, radical formation as a consequence of cavitation bubble collapse, or increased interfacial area arising from the creation of cavitation bubbles in solution), while concave effects are influenced by multiple phenomena (*e.g.*, increased fluid shear arising from either cavitation or streaming. General trends between frequency and the ultrasonic phenomena (shown in Fig. 1) are discussed in more details for each mechanism in Sections 4.1–4.3 (*vide supra*).

## 5 The effects of ultrasound on solids’ processing

In this section the various ultrasonic effects and mechanisms are discussed in greater detail, following the framework proposed in Section 4 and illustrated in Fig. 2. Ultrasound is shown as a unique method to holistically tailor the product properties by exploiting different mechanisms. This analysis is grounded in numerous case studies (from different material classes) from the literature. Herein, a distinction is made between the formation stage (*i.e.*, the synthesis or assembly step) and the post-treatment step. This does not necessarily imply that the distinction between these two steps is always clear-cut, or that the same ultrasonic mechanism cannot influence both synthesis and post-treatment in different ways.

### 5.1 Formation stage

The formation or synthesis of solid materials can be affected by chemical, mechanical, and transport effects, which can occur simultaneously.

#### 5.1.1 Chemical effects: radical formation

Sonochemical radicals that appear in solution can either recombine and disappear, such that there is no chemical effect, or drive radical-induced reactions. If oxidizers or reducers are present in solution, their effect can be multiplied through the generation of secondary radicals.^85^ There is no single parameter that can assess a material's willingness to interact chemically with radicals. Some measurements, such as reaction mechanism studies, can be conducted to obtain an idea, but it remains questionable whether such tests provide sufficient insight to estimate the chemical effects ultrasound will induce in the system. From a molecular point of view, increased interaction with radicals occurs when the original spin-paired molecule is capable of better stabilizing the radical. Important properties are the solid molecules polarity, steric hindrance, electron delocalization, potential to be a proton donor, and bond energies. However, there are also examples where, under sonolysis, the cleaved bond is not always the least stable one: in sonochemical hydrostannylations the Sn–H (bond energy *ca.* 310 kJ mol^−1^) is cleaved over the Sn–C bond (*ca.* 250 kJ mol^−1^).^132^

**Fig. 4:**
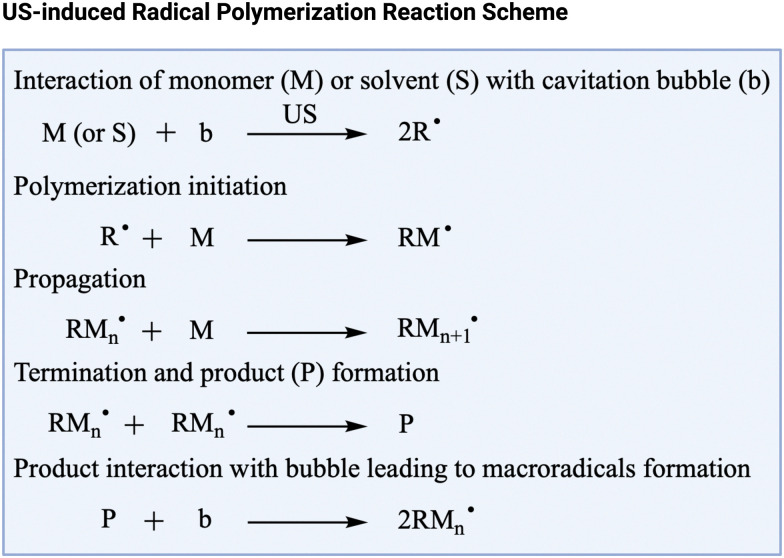
Simplified scheme for reactions involved in ultrasonic radical polymerization. US = ultrasound. Adapted from McKenzie *et al.*^20^ Created with https://Biorender.com.

Solids that are formed through radical-induced reactions can be affected by ultrasonically produced radicals. A notable example is that of polymers that are formed through addition (also called chain) polymerization. This process entails the sequential addition of monomers as a result of a free radical reaction. In conventional addition polymerization reactions, the free radicals originate from thermal or photochemical decomposition of an initiator,^161–163^ or the pure monomer.^164^ The formation of solvent-derived radicals during sonoprocessing provides an alternative to relying on the presence or the external addition of these initiators.^20^ For instance, during the ultrasound-induced polymerization of acrylonitrile in water using a sonicator operating at a frequency range from 400 to 1500 kHz and power intensities up to 10 W cm^−2^, water dissociation produces the free radicals that initiate the polymerization.^165^ A general representation of the ultrasonic radical polymerization mechanism is presented in Fig. 4. In cases where the solvent fails to produce an adequate number of radicals, the introduction of a small quantity of water (1.2 wt%) can effectively trigger the polymerization, as illustrated during the ultrasound-induced polymerization of diallyl terephthalate using a sonotrode (at 25 kHz and 2.5 W g^−1^ of solution).^166^ In another example, the radicals generated by sonication have proven advantageous for reversible deactivation radical polymerization (RDRP), a relatively recent polymerization technique.^20,21^ Reversible addition–fragmentation polymerization (RAFT), a primary category within RDRP, has been initiated by ultrasonically produced radicals in both aqueous^167^ and organic solvents.^168^ Notably, ultrasound-assisted RAFT or sonoRAFT polymerization achieved up to 90% monomer conversion (414 kHz, up to 2.5 W cm^−2^) in aqueous solutions of acrylate containing a RAFT agent.^167^ Moreover, sonoRAFT (490 kHz, 2 W cm^−2^) of acrylates and acrylamides was initiated by DMF and DMAc radicals, obviating completely the need for external initiators or additives.^168^

**Fig. 5:**
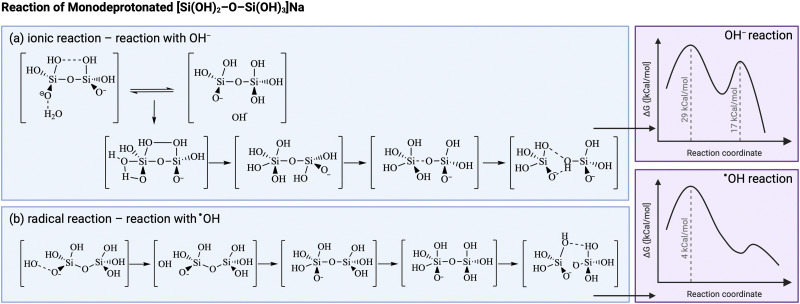
Comparison between (a) ionic and (b) radical reaction kinetic pathway for silica condensation during zeolite synthesis. The presence of radicals (from sources other than ultrasound) is thought to modify the intermediates, hence to lower the energy barrier. Adapted from Feng *et al.*^172^ Created with https://Biorender.com.

Another illustrative example is that of sonication during the production of zeolites. Radicals are thought to accelerate the dissociation of Si–O bonds in the aluminosilicate gel and polymerization of Si–O–Si bonds in the nucleation stage in alkaline media.^169–174^ Several studies use density functional theoretical (DFT) calculations to get insights on the molecular structures involved,^172,175–177^ however true mechanistic understanding remains challenging. Furthermore, it must be stressed that in these studies the OH radicals are generated by different means than sonication, hence the true contribution of ultrasonically-generated radicals compared to other effects of ultrasound occurring during zeolite synthesis still needs to be fully confirmed.^178^ Conventional aluminosilicate formation in alkaline medium is believed to proceed through a two-step formation mechanism: the first transition state comprises the formation of a O_3_Si_2_ complex, which creates a pentacoordinated Si intermediate, as shown in Fig. 5. This short-lived intermediate then goes through the second transition state, with the removal of a water molecule to form the product.^170,179^ In the case of Al–O–Si bond formation, there is an additional initial step of water-mediated proton transfer between the Si(OH)_4_ and the Al(OH)_3_^−^, which makes it the first transition state, followed by Al–O–Si dimer formation in the second transition state.^179^ DFT calculations report a preferential acceleration of Si–O–Si bonds compared to Al–O–Si bonds in presence of radicals, hence possibly increasing the Si/Al ratio in the zeolite product.^176^ Gibbs-free energy calculations of the Si–O–Si process in presence of OH ions or OH radicals show a similar mechanism as the one explained above, however, both activation energies to the transition states are much lower in the radical-driven process, meaning that the presence of OH radicals will speed up both the depolymerization and recondensation of silica.^172^ It is possible to discern the resemblances to polymers, with the silica undergoing polymerization to form the solid product.

In some cases, the appearance of radicals may have detrimental effects on the formation stage,^9,89,180^ emphasizing the need to determine the optimum sonication time during the nucleation or synthesis stage. For instance, ultrasonication during organic solid synthesis processes may result in the chemical degradation of the organic compound (*e.g.*, paracetamol, mefanamic acid, levodopa^89,180,181^), which reduces the purity of the product. Usually, the highest rate of degradation for organics in aqueous solutions occurs at the gas–liquid interface, due to a high steady state concentration of OH radicals in this region. Such amphiphilic molecules (*i.e.*, hydrophilic hydrocarbons) are surface-active, as their hydrophobic part typically remains close to the surface and is not dissolved in an aqueous environment. For this reason, the concentration of these compounds will be higher at the interface, therefore the OH radicals will attack the compound instead of recombining to give H_2_O_2_.^9,182,183^ Jordens *et al.*, for instance, investigated the effect of ultrasonic frequency (41–1140 kHz) on the sonochemical degradation of paracetamol in water at a constant calorimetric power (0.053 W mL^−1^) over 88 minutes of sonication.^89^ They found that paracetamol degradation occurred at all studied frequencies, with minimal degradation (6%) at the lowest frequency (*i.e.*, 41 kHz) and maximum degradations of 65% and 52% at 165 kHz and 850 kHz, respectively. In the presence of a radical scavengers (1-butanol, a scavenger in the gas–liquid interfacial region), the degradation dropped to an insignificant level regardless of frequency.^89^ Isariebel and coworkers compared the degradation rates of levodopa and paracetamol in water mixtures when sonicating at 574, 860, and 1134 kHz, with the latter showing the lowest degree of degradation.^181^ For high concentrations (150 mg L^−1^), degradation percentages of 61% and 73% were obtained, respectively (after 8h at a calorimetric power of 0.30 W mL^−1^ and 574 kHz), whereas for lower concentrations (25 mg mL^−1^) 100% degradation of both compounds was reached.^181^ Increasing the calorimetric power from 0.03 W mL^−1^ to 0.10 W mL^−1^ (also at a frequency of 574 kHz) increased the degradation rate of paracetamol from less than 0.05 to 0.30 mg L^−1^ min^−1^.^181^ Degradation in the presence of H_2_O_2_ (a radical promoter or scavenger, depending on the conditions) improved degradation provided the correct concentration was used.^181^ In the presence of 1-butanol, they also found an inhibition of degradation for both compounds.^181^ However, 17% degradation was still achieved for levodopa (compared to 5% for paracetamol), indicating more substantial degradation occurring in the bulk phase due to levodopa's higher hydrophilicity compared to paracetamol. Sharma and Gogate showed a decrease in mefenamic acid yield (which can be attributed to radical-induced degradation) when sonicating longer than 10 minutes at 40 kHz and 0.5 W mL^−1^.^180^ When celecoxib melt crystallization was investigated, it was found that pulsed sonication for one minute at 80% amplitude with a sonotrode at 24 kHz and 8 W mL^−1^, was not enough to detect any degradation of the crystallized drug.^184^

Based on these reports, some general trends for sonochemical solid degradation through radical chain reactions emerge: Degradation increases with both power and sonication time due to the generation of more radicals. An intermediate maximum degradation occurs with frequency variation,^89^ corresponding to the optimal frequency for radical generation (see also Fig. 3). Higher initial solid concentrations lead to lower degradation percentages,^181^ as more radicals are required to obtain similar degradation effect.^181^ The presence of radical scavengers significantly reduces degradation by neutralizing the radicals in the gas–liquid interface region.^89,181^

In other cases, ultrasound-induced degradation may be desirable, for instance for the degradation of pollutants in water streams.^128,185^

#### 5.1.2 Mechanical effects: increased fluid shear

Ultrasonication can significantly enhance fluid shear in a system, primarily through the collapse of cavitation bubbles in the LFUS regime and through acoustic streaming in the HFUS regime. The resulting solute-solute molecular collisions can impact or induce the formation or synthesis of new solid material.

Enhanced fluid shear has, for example, been shown to considerably increase primary nucleation rates for organic cooling crystallization from solution (*e.g.*, for glycine,^186^ butyl paraben,^187^ and paracetamol^67,188^), which is reflected in a drastic reduction of the metastable zone width (for polythermal measurements) or shorter induction times (for isothermal measurements).^189^ Similarly, in evaporative, antisolvent, and reactive crystallization processes increased fluid shear leads to an increase in the driving force for crystallization.^190^ Although it is still contested and subject of debate whether the enhanced fluid shear mechanism is the precise cause of ultrasonication increasing the primary nucleation kinetics with several orders of magnitude^7,9,11,13,150^ (as reported in literature: *e.g.*, ref. 67, 155, 191 and 192), Jordens *et al.* identified it (in their case called ‘flow induced nucleation’) as the most likely theory.^9^ This theory is further supported by recent findings from Devos *et al.*, who demonstrated that the primary nucleation rate of paracetamol from aqueous solution could be precisely controlled in the acoustic streaming regime by adjusting the acoustic power.^67^ By varying the streaming conditions (such as frequency, from 704 to 1344 and 2304 kHz, or power densities from 0 to 40 W mL^−1^), fluid shear could be controlled (between 0 and 15 s^−1^) without inducing cavitation.^67^ Other theories aiming to explain the effects of ultrasonication on crystallization are discussed in Sections 5.1.3–5.1.6. The increased nucleation rates due to sonication compared to silent conditions have also been exploited to promote the appearance of kinetically favored polymorphs.^9,13,190^ However, in some cases, also other mechanisms to explain the appearance of metastable polymorphs have been proposed:^193,194^ For example, pulsed sonication (at 20 kHz and intensities between 1.4 and 15 W cm^−2^) was found to favor the appearance of the β-form of *p*-aminobenzoic acid, which was hypothesized to result from ultrasound-induced changes in solution clustering prior to nucleation.^194^

The impact of sonication on crystal growth rates has received comparatively less attention, leaving its effects less well-understood,^9,13^ but also for crystal growth increased fluid shear can impact the kinetics. Based on measurements of nucleation and growth rates for antisolvent crystallization of KCl with mechanical stirring (at 250 rpm) and sonication (at 20 kHz and 500 W), Nalajala and Moholkar determined that sonication significantly increases nucleation rates while (slightly) reducing growth rates compared to mechanical agitation.^110^ By coupling these experiments to bubble dynamics simulations, they posit that the shockwaves increase the nucleation rate and that the microturbulences govern the growth rates.^110^ For brittle materials, the opposing effect of fragmentation (discussed further) must be considered alongside growth. This entanglement of phenomena complicates both understanding and quantification of the mechanisms. Either way, it can be asserted that sonication affects the growth rate of crystals in a similar manner as stirring.^11^

The mechanical effects of ultrasonication are also harnessed during the pretreatment stage of zeolite synthesis. Particularly in hydrothermal batch processes, which are generally constrained by mass transfer, the application of ultrasound can increase the crystal yield.^195^ The enhanced mixing and formation of secondary flows due to bubble collapse promote the dispersion of amorphous reactant mixture with precursor particles,^196^ which increases the crystallization rate. Chen and coworkers observed a decrease of zeolite ZSM-5 particle size under increasing sonication powers (97, 194, 323 W).^197^ Ultrasound was delivered indirectly by an ultrasonic horn operating at 24 kHz immersed in a water bath, together with 4.4 g of reactant mixture in a 7 mL polymethylpentene vial. At 194 and 323 W, the crystal size distribution decreased from an average of 700 nm to 200 nm for a nearly constant yield (always between 12 and 13%) at the end of the crystallization process, suggesting that higher powers lead to an increase in the nucleation rate. However, at 97 W, no appreciable reduction in size was observed compared to the silent case, but a decrease in crystallization time, suggesting an enhancement in the growth rate thanks to ultrasonication.^197^ Similar results were observed by Andaç *et al.* when using a conventional ultrasonic bath (35 kHz, no power reported) for zeolite A synthesis: in this case not only crystallization rates but also yield increased under sonicated conditions.^195^ The application of ultrasound during pretreatment of MOR zeolite (ultrasonic bath, 37 kHz, around 0.5 W mL^−1^–50 W per 100 g reactant mixture) has even been reported to affect the distribution of Si and Al atoms in the zeolitic frameworks.^198^ It is hypothesized that the shockwaves induce the disintegration of the Si source, which results in faster dissolution and enables Al distancing in the product framework. Moreover, ultrasonically synthesized MOR zeolites were found to have more accessible microporosity (in as-made form) compared to conventional mechanical stirring.^199^ The effects during polymerization are comparable: improved mass transfer and catalyst dispersion.^1,200^ This results in a higher polymerization rate, as well as the synthesis of polymers with higher molecular weights and reduced polydispersity.

#### 5.1.3 Mechanical effects: fragmentation

In principle, fragmentation occurs following the formation or synthesis step as it requires the presence of sufficiently large solid particles (see Section 5.2.2). However, there are instances where fragmentation of newly formed or a-priori added solids can coincide with particle formation, catalyzing the process as these formed fragments act as seed particles. The possible molecular mechanisms underlying this effect are discussed in Section 5.2.2. One relevant example is the ultrasound-induced fragmentation of seed crystals to promote secondary nucleation^9,11,13,155^ by increasing the available seed area for secondary nucleation.^13,201^ The newly formed fragments then act as new nucleation sites.^9,190,202^ Even in the absence of seed crystals, fragmentation might be important: regardless of the dominant mechanism for nucleation of the first primary crystal (*i.e.*, in the absence of seeds) ultrasound-induced secondary nucleation often becomes the dominant mechanism after the appearance of the first crystal(s). For ultrasonication (at 30 kHz and 0.04 W mL^−1^) during cooling crystallization of paracetamol at various stages (before nucleation, during desaturation, and at complete desupersaturation) it was implied that secondary nucleation was dominant after the appearance of the first crystal.^203^ This is well in line with the observation that sonication can be a viable alternative to seeding.^155,204^ The appearance and subsequent attrition of a single (parental) crystal which catalyzes the generation of new (secondary) crystals has been hypothesized to be the dominant nucleation mechanism in industrial crystallizers (a phenomenon known as the single nucleation mechanism) even in the absence of ultrasound.^202,205,206^ As such, it seems this ultrasonic fragmentation mechanism can be leveraged in various industrial crystallization processes.

The fragmentation mechanism of ultrasound can also be exploited for the production of enantiomerically pure crystals from a racemic solution with a large crystal enantiomeric excess (*e.g.*, ref. 207–209). Recent studies have demonstrated that ultrasound-induced fragmentation can be a faster alternative compared to more conventional deracemization *via* attrition-enhanced mechanical grinding in solution using glass beads,^207,210^ known as Viedma ripening.^210^ The small fragmented crystals either dissolve (due to the Gibbs-Thomson effect), allowing larger crystals to grow at their expense, or incorporate into crystals of the same chirality (chiral recognition).^211^ This reincorporation is favorable for the enantiomer in excess, such that an enantiomerically pure product can be obtained autocatalytically.^211^ While the experimental results suggest that the fragmentation mechanism is important, it is worth pointing out that also the local hotspots generated by the cavitation bubble implosion may play a role in the deracemization process through cyclic dissolution and recrystallization.^15,207^

The fragmentation (also referred to as degradation, without necessarily implying chemical degradation) of monomers, macromolecules or other compounds can also be used to increase the polymerization kinetics. The mechanical cleavages of the molecular bonds result in the generation of free radicals – of a different type than the radicals originating from solvent splitting (discussed in Section 5.1.1) – that effectively initiate the polymerization. Examples of such ultrasound-induced chain polymerization reactions include the production of poly(methyl methacrylate) (PMMA, sonication at 20 kHz and power intensity up to 27 W cm^−2^)^212^ and polyvinylpyrrolidone (sonication at 20 or 40 kHz and power density of 12 W mL^−1^, or at 540 kHz and power density of 1.6 W mL^−1^).^213^ Additionally, ring-opening polymerization, which entails the reaction between a ring-containing monomer and a polymeric chain ending with either an anion or cation,^163,214^ can follow this mechanism. The ultrasound-assisted synthesis of cyclic bisphenol A (at 20 kHz and up to 430 W g^−1^ of monomer, both with and without the use of initiators) is an example.^215^ In this case, sonication could generate strong shear forces capable of breaking cyclic bonds. Nevertheless, it was noted that this mechanism is unlikely to occur for the monomers used, and an alternative hypothesis was proposed. This alternative hypothesis states that it is the presence of impurities in the bulk (such as NaOH, NaCl or Na_2_CO_3_) which acts as nucleophilic initiators for ring-opening polymerization. The application of ultrasound resulted in reduced processing times, lower reaction temperature requirements, and accelerated molecular weight increase.^215^

#### 5.1.4 Transport effects: bulk temperature increase, local temperature and pressure hotspots

One of the most noticeable effects in sonicated systems is the bulk temperature increase. The extent to which ultrasonication increases the bulk temperature is not always reported and depends significantly on the efficiency of the reactor's temperature-control system and the reactor scale. Quantifying this (non-linear) heat increase is best achieved through experimental testing. Sesis *et al.* report a temperature increase of 0.02 °C s^−1^ at 0.40 W mL^−1^ nominal power (or 0.084 W mL^−1^ calorimetric power) and 0.03 °C s^−1^ at 0.8 W mL^−1^ nominal power (corresponding to 0.124 W mL^−1^ calorimetric power) before reaching a plateau for sonication at 20 kHz in a reactor vessel with 250 mL of water. In comparison in a vessel with 25 L of water, they report a temperature increase of 1.3 × 10^−4^ °C s^−1^ and 1.8 × 10^−4^ °C s^−1^, for sonication at 0.4 × 10^−3^ W mL^−1^ and 0.8 × 10^−3^ W mL^−1^, respectively. Pal *et al.* measured an increase of 50 °C in 3 h of sonication with a ultrasonic probe at 150 W input power (corresponding to a temperature ramp of around 0.005 °C s^−1^) when pretreating the reactant gel for zeolite synthesis.^171^

A bulk temperature increase can change the driving force of solids formation. For instance, in cooling crystallization raising the temperature reduces the process kinetics. However, the mechanism influencing an increase in nucleation and growth rates tend to override this counteracting bulk temperature effect. For solid formation processes conducted at high temperature (*e.g.*, zeolites), the bulk temperature increase is negligible. Increased temperatures due to ultrasonication enhance the reaction rates of most polymerization reactions. For instance, for several polymers such as polyurethane and poly(ε-caprolactone) higher temperatures due to sonication were attributed to have an effect on the reaction rate,^216,217^ resulting in higher molecular weights and dispersity values. It must be acknowledged, though, that these observations were probably mainly affected by the improved mass transfer of the reactants and dispersion of the catalysts in the viscous monomer solution (see Section 5.1.2) as described in Fig. 2(C).

It is worth noting that an increase in the bulk temperature also affects the cavitation behavior in solution.^218^ Temperatures closer to the boiling point of the solute increase the vapour content of the bubbles, which cushions cavitation bubble implosion.^178^

As mentioned in Section 3.3, cavitation bubble implosion creates localized temperature and pressure hotspots in solution. However, the temperature effect of hotspots is typically difficult to discern from the bulk temperature increase. Specifically, the synthesis of zeolites can be taken as a characteristic example of decoupling the bulk temperature increase and the presence of local hotspots. While high-temperature syntheses do not experience advantages from overall temperature increases attributed to ultrasonic energy dissipation, the existence of localized hotspots can introduce significant alterations to the synthesis mechanism at a molecular level, as highlighted earlier. This hypothesis was supported by applying ultrasonication during a pre-treatment step for the synthesis of NaP zeolite crystals conducted at room temperature (30 °C in this case), where the only temperature increase (up to 80 °C) was caused by ultrasonication (at 20 kHz and 150 W),^171^ followed by low temperature synthesis. The obtained product appeared to have a lower number of silanol structural defects compared to conventional stirred aging, and a different Al distribution.^199^ Furthermore, localized hotspots are claimed to be the reason for kinetics enhancement in the case of MOF synthesis conducted at constant bulk temperature under ultrasonication.^219^ The observed increase in the Arrhenius pre-exponential factor supports the hypothesis of increased kinetics caused by transport effects rather than chemical effects (which should lead to a decrease in the activation energy).^219,220^

When considering hotspots, the focus usually centers on the effects of high temperatures. A theoretical study by Hickling posits that it is actually the fast cooling following the bubble collapse which affects the formation of solids.^221^ It is, for example, hypothesized that during crystallite reduction (as discussed in Section 5.2.2 for Al_3_Ni_2_ due to ultrasound-induced interparticle collisions), not only the melting but also the rapid cooling (for solidification) is crucial.^222^ Jordens *et al.* concluded in their analysis that the effect is probably too small to affect the rate of typical crystallization processes.^9,154^

If the solubility of a solid is high and the dissolution kinetics is fast, solids may dissolve after formation in the vicinity of temperature hotspots. For inorganic aluminosilicate synthesis, the concentration of the soluble silicate species in the liquid phase increases as a result of a temperature increase. As a result of this, the Si/Al ratio is higher for products pre-treated with ultrasound,^223–225^ and a higher driving force is obtained.^2,226^ During the nucleation of crystals, these local hotspots are thought to improve the purity of the product, based on the somewhat questionable (considering their short duration and spatial randomness^14^) hypothesis that redissolution of impurities at the local hotspots occurs.^227^ For polymers with a low degradation temperature, the formation of local hotspots after transient cavitation bubbles' collapse during chain polymerization in nonaqueous solvents leads to pyrolytic degradation.^228,229^

Lastly, there is also a sudden pressure variation (up to 1000 atm^96^) accompanying cavitation implosion. Besides its mechanical effects, there are hypotheses that suggest that the sudden pressure change itself impacts the thermodynamic equilibrium of the solid phase.^150^ In the case of crystallization, this could lead to a scenario in which the nucleation sites experience a considerably higher driving force, albeit for only a nanosecond, potentially triggering nucleation.^230^ However, when Harzali *et al.* investigated ultrasound-induced nucleation of ZnSO_4_·7H_2_O in water (for which the solubility is independent of pressure),^150^ they still observed increased nucleation kinetics under strong sonication for 720 s (at 20 kHz with power densities of 0.03 and 0.06 W mL^−1^), which shows that that pressure swings due to cavitation implosion do not affect the driving force to such an extent that it impacts nucleation.^150^

#### 5.1.5 Transport effects: local concentration gradients

As discussed in Section 4.3, the rapid collapse of cavitation bubbles is thought to cause concentration gradients (as a result of pressure-diffusion) in the region around the implosion provided that the solute density is higher than the solution density (segregation hypothesis, see Section 4.3),^9,150^ which in turn would increase the kinetics of solid formation (synthesis or nucleation).

Analytical calculations^150,153^ have shown that the segregation hypothesis could be a plausible explanation, but sonication (at 41 kHz and 53 W L^−1^) during crystallization experiments of paracetamol (density higher than water) and 3,5 dimethylpyrazole (density lower than water) in water showed a reduction in the induction times for both compounds.^9^ Regardless, it remains sensible to continue considering this hypothesis, with further investigations into organic and inorganic crystallization needed to better understand the validity of the segregation hypothesis.

#### 5.1.6 Transport effects: presence of interfacial area

In the frequency range between 20 kHz and 500–600 kHz (see Fig. 3), the presence of cavitation bubbles increases the interfacial area within the solution. Since some solids adhere preferentially to a bubble's surface to minimize their interfacial energy, this phenomenon can influence the formation and synthesis of solids.

In organic crystallization the presence of gas bubbles has been shown to increase the nucleation kinetics, as the bubbles can act as active nucleation centers that promote localized nucleation.^155–157,231^ The importance of this phenomenon in the context of sonocrystallization remains unclear. An analysis of the classical nucleation theory parameters for paracetamol nucleation from aqueous solution shows, however, that when ultrasonication is applied (at 30 kHz and 1.42 W mL^−1^ until primary nucleation is detected) the effective interfacial energy is lowered considerably (assuming that growth mechanism is not impacted).^155^ These results suggest that the mere presence of cavitation bubbles significantly impacts primary nucleation. The presence of additional interfacial area is also thought to impact the ultrasonic (for 60 min, with an ultrasonic bath working at 40 kHz and 50 W) pre-treatment of reactant mixture for the synthesis of MCM-49 mesoporous silica materials.^232^

The presence of bubbles can also cause buoyancy effects (in large reactors), causing the smallest (newly formed) particles to rise to the surface (*i.e.*, ultrasound flotation). This results in their removal from the processing effects applied to the rest of the bulk. This effect can also be exploited, for example during mineral flotation to increase the process efficiency (through cavitation and acoustic radiation).^233^

Lastly, ultrasound is believed to influence the reactor wall interfacial area by generating so-called container imperfections,^234^ which may, in turn, impact the solid formation process.

### 5.2 Post-treatment stage

Ultrasonication affects solid materials not only during their synthesis, but also when they are already present in the solution under sonication. First, heterogeneous sonochemistry is discussed, which covers the modification and activation of otherwise inert compounds, as a result of ultrasonic chemical effects. This is followed by a description of experimental studies demonstrating the different mechanical effects (as discussed in Section 4) that can occur during the post-treatment stage. Foremost among the mechanical effects are fragmentation and deagglomeration, arising from collisions induced by shockwaves and microjets within the solution. The modification of the material itself on a molecular level, leading to a structural modification of the as-synthesized solid is also touched upon. Lastly, the transport effects arising from the presence of solids within a reacting solution (*i.e.*, heterogeneous sonocatalysis) under sonication are presented.

#### 5.2.1 Chemical effects: sonochemistry in solid–liquid systems

Sonochemistry within heterogeneous solid–liquid systems involves two effects: sonochemical switching and/or solid surface activation (see Section 5.2.3). Several examples of reaction pathway shift due to ultrasonication can be found for homogeneous or heterogeneous liquid–liquid reactions, which are outside the scope of this review.^3,235,236^ Only few examples, however, report this phenomenon in solid–liquid systems.^237–240^ The most prominent one comes from Ando and coworkers, who applied ultrasound on different mixtures of aromatic solvents and substituted benzyl bromides in the presence of solid potassium cyanide (KCN) and solid alumina powder, and showed that a different chemical pathway took place.^237^ Under mechanical agitation, the resulting reaction is a Friedel–Crafts electrophilic substitution that yields diphenylmethane derivatives. However, when the mixture is pre-sonicated at 45 kHz and 40 W mL^−1^, for 3 h, the reactants undergo nucleophilic substitution to give benzyl cyanide.^237^ They proposed that the alumina acid sites, known to catalyze the Friedel–Crafts reaction, are attacked by KCN *via* enhanced solid–solid contact under ultrasonic irradiation. As a result, the basic sites of alumina become available for nucleophilic substitution.^237,241^ A different mechanistic theory is proposed by Vinatoru and Mason, who suggest that the remaining water on the alumina surface provides electrons to ionize KCN and promote nucleophilic reaction *via* electron transfer mechanism.^242^

#### 5.2.2 Mechanical effects: fragmentation and deagglomeration

Sonofragmentation is often a dominant effect during the post-processing. It has been well-studied for a large variety of brittle (both inorganic^98,243,244^ and organic^146,245^) compounds. It is hypothesized that the activation energy to break a particle correlates to the solids’ binding energy.^146^ As a consequence, materials with stronger molecular bonds in the structure will break less easily than materials with weaker bonds, but also the presence of defects on the surface can trigger particle fragmentation. Furthermore, also the solid morphology plays an important role during the fragmentation.^9^

**Fig. 6:**
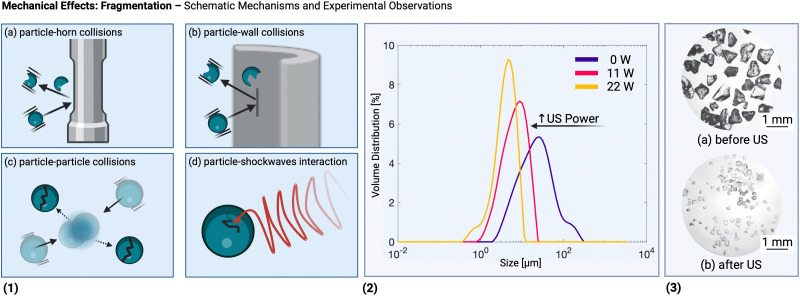
(1) Different solid particle fragmentation mechanisms, ranked in decreasing order (from (a)–(d)) according to the force (*i.e.*, impact) required to induce fragmentation. (2) The effect of sonication at different powers on the volume-based size distribution of barium sulfate particles in aqueous solution flowing through a microreactor connected to a Langevin transducer (operated at 21 kHz). Adapted from Delacour *et al.*^246^ (3) Microscopy pictures of NaF crystals before and after sonofragmentation: sonication for 900 s of a 0.2 wt% slurry with an ultrasonic horn (10 W cm^−2^ at 20 kHz). US = ultrasound. Adapted from Kim and Suslick^146^ with permission from John Wiley and Sons. Created with https://Biorender.com.

For low molecular weight solids, a number of studies have been conducted to uncover whether the dominant fragmentation mechanism is due to particle–transducer, particle–wall, particle–particle, or particle–shockwave interactions (see Fig. 6(1)). By decoupling experiments in which the particles were prevented from hitting the reactor wall by suspending them in a flexible membrane^146,245^ and kinetic experiments, Zeiger and Suslick showed that particle–shockwave interactions are the dominant mechanism for fragmentation of molecular crystals.^245^ Experiments were performed using an aspirin slurry as a model system suspended in dodecane (in which aspirin is not soluble) sonicated at 20 kHz with an ultrasonic horn (at calorimetric powers of 0.16 to 3.05 W mL^−1^ for 30 s of sonication).^245^ Similarly, Kim and Suslick showed experimentally that sonofragmentation (for 140 s of sonication, at 20 kHz, and 10 W) of alkali halides solids (*i.e.*, ionic crystals, like NaF, LiCl, NaCl, *etc.*) is also dominated by the interaction between crystals and shockwaves.^146^ As in those experiments interparticle collisions did not contribute considerably to the fragmentation, increasing the slurry concentration did not impact the results.^146,245^Fig. 6(3) shows a representative example of such crystals before and after sonofragmentation.

**Fig. 7:**
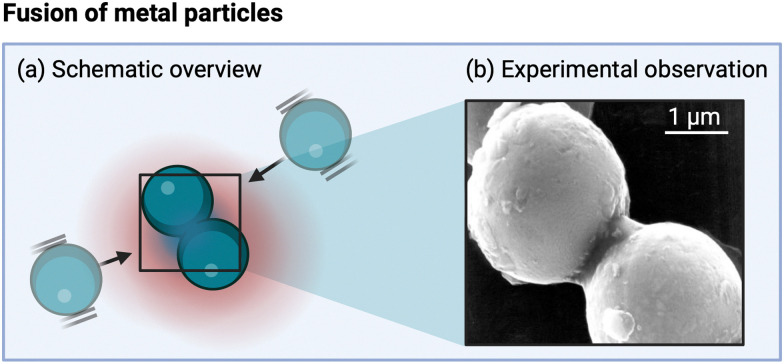
Fusion of metal particles: (a) schematically shown, (b) scanning electron microscopy (SEM) image of Zn interparticle collision, resulting from the shockwaves produced by the collapse of cavitation bubbles during sonication. A neck is formed between two particles at the point of impact due to high-velocity collisions (*i.e.*, interparticle melting). The powder was sonicated for 30 min in pure decane with a horn at 20 kHz and 50 W cm^−2^. Similar neck formation was observed for sonicated slurries of Sn and Fe particles.^98^ Adapted from Doktycz and Suslick.^98^ Reprinted with permission from AAAS. Created with https://Biorender.com.

For inorganic compounds research has shown that interparticle collisions are dominant.^98^ Doktycz and Suslick studied the effect of high power ultrasound (50 W m^−2^) in metal powder slurries (*i.e.*, Zn, Ni, Cr, and Mo) in hydrocarbon liquids, and found that they are affected by the shockwaves caused by the bubble collapse itself.^98^ As a consequence, particles collide with extreme velocities, in areas of localized heat. If their melting temperature is lower than the local temperature created by the collapse, particles are even found to melt (such interparticle melting does not strictly fall under sonofragmentation, but can be interpreted within this category), as shown in Fig. 7.^98^ The conditions for metal interparticle melting are dependent on the solid concentration and sonicated liquid medium.^222,247^ Cherepanov *et al.* found that by increasing Al_3_Ni_2_ concentration in suspension, the intermetallic crystallite size grew as a consequence of more frequent interparticle collisions (due to sonication for 60 min at 20 kHz with an ultrasonic horn and 140 W cm^−2^ of power). This is similar to subjecting the crystals to particle annealing at 580 °C for 2 h, followed by fast cooling. After reaching a maximum size at 10 wt%, the crystallite dimensions started to decrease, suggesting that the ultrasound-induced collisions where so frequent that they reached a local temperature higher than the melting temperature.^222^ Also Gao *et al.* found that sonofragmentation of silica particles of around 100 μm (with a frequency of 20 kHz and 0 to 300 W cm^−2^) could be mimicked by conventional stirring, which supports the hypothesis that the particle–shockwaves interaction is not the dominant mechanism in their experiment.^248^

Regardless of the precise mechanism through which sonofragmentation occurs (shown in Fig. 6) cavitation implosions are clearly the driving cause. Some research has been devoted to the role of typical ultrasonic parameters on the fragmentation behavior. Jordens *et al.* fragmented paracetamol crystals in water over a broad frequency range (from 30 to 1140 kHz, at a calorimetric power of 0.13 W mL^−1^) for 180 minutes.^94^ At frequencies of 166 kHz or lower, the particle size reduced considerably – *e.g.*, from 65 to a limit or threshold size of 35 μm.^94^ Such threshold sizes have also been observed for other compounds in literature: *e.g.*, for sodium chloride^249^ and manganese carbonate.^250^ At higher frequencies the particle size did not change as much.^94^ Similar observations were made during the cooling crystallization of *p*-Aminobenzoic acid from ethanol,^251^ albeit at different frequencies (22 kHz to 1 MHz and 0.003 to 0.12 W mL^−1^). While the authors attributed the observed differences in frequencies primarily to variations in crystal shape and hardness,^251^ differences in the system setup may also contribute. The crystal length decreased with sonication at 20 kHz, while only minor changes were observed for sonication between 200 kHz and 2 MHz.^251^ In general, this behavior can be attributed to the implosion behavior of cavitation bubbles, which is more pronounced at low frequencies due to the cavitation bubble size being larger.^4^ As higher ultrasonic powers result in more violent implosions,^4^ the sonofragmentation rates are larger and the limit threshold size is achieved faster.^94^ Under some specific experimental conditions, *e.g.*, frequencies close to 20 kHz, the threshold fragmentation size can be reduced.^94^ Raman and Abbas found that for sonofragmentation of aluminium oxide particles an optimal temperature range exists, where the cavitation implosions are strongest.^244^

Others have investigated the extent to which sonofragmentation can be exploited during post-processing of solids, leading to a variety of applications. For instance, the sono-post-processing of reactive metal powders exploits interparticle collisions to improve their activity in metal-catalyzed reactions. Sideways collisions have been identified as the cause of removing a passivating oxidized layer, responsible for the deactivation of the catalytic powder.^63^ The most common application of sonofragmentation is the processing of pharmaceutical compounds either during the formation or during the post-processing step, to reduce the particle size and size distribution.^9,13,94^ Experiments with adipic acid, for example, illustrated that sonication (at 20 kHz at a power of 82 to 95 W/100 g of slurry) resulted in the production of crystals with a similar size as micronization.^252^

Although porous materials tend to be minimally affected or remain completely unaffected by sonofragmentation of the crystals, due to their hardness combined with their generally smaller size (often in the sub-micron range),^226^ ultrasound remains a highly valuable technique to deagglomerate particle clusters to their primary particle size, through breakage of weak interparticle bonds.^64,143,253^ For example, Kusters *et al.* demonstrated that ultrasonic deagglomeration in solution can be equally effective as conventional dry grinding methods in producing sub-micron deagglomerated primary (*e.g.*, silica or zirconia) particles by applying the same or lower energy input.^143^ They tested powers between 2.5 W and 100 W at 20 kHz on solutions between 40 and 120 mL with variable solid concentration. Ultrasonic deagglomeration could be attributed to the interplay of interactions between particles, shockwaves, and microjets in the solution. This was concluded based on the observation that the fragmentation rate only became dependent on particle concentration above 50 wt%, when the slurry was too concentrated and therefore not sufficient cavitation zones were created.^143^ No size reduction was detected for titania primary particles below 1 μm subjected to sonication (at 20 kHz), as a result of the strong intraparticle bonds in metal oxides.^253^

Also polymer fragmentation (which will be referred to as “degradation” for coherence within the polymer field, but not to be confused with sonochemical or pyrolytic degradation) has been the subject of numerous studies aimed at investigating the effects of ultrasound in the presence of both organic and aqueous solvents.^20,23^ Long polymeric chains are fractured and smaller chains are formed until a minimum molecular weight (*M*_w_) is attained. Beyond this point, in analogy to the threshold size commonly observed for fragmentation of large crystals, further reduction in molecular weight is impossible.^22,40,254^ Some studies have shown that when cavitation is suppressed, polymeric degradation is seriously limited.^23,255^ However, other studies reported degradation in complete absence of cavitation when starting from high *M*_w_ polymers.^256–259^ In the case of dilute solutions of PMMA and polystyrene for instance, slow degradation rates were observed in the absence of cavitation, leading to the conclusion that molecular fission occurred. No specific trends (*vide infra* – Gooberman hypothesis) related to the size of the degraded molecules were reported.^256^

**Fig. 8:**
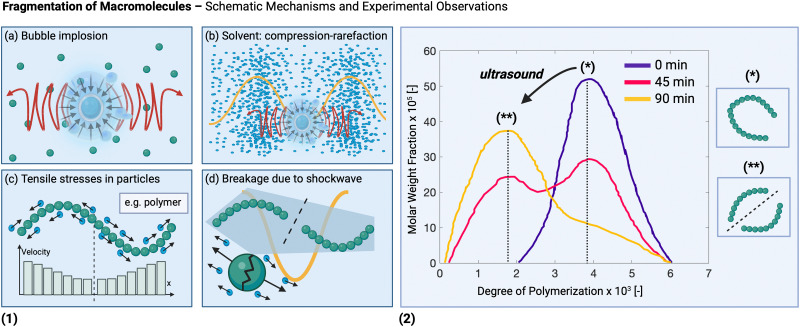
Fragmentation of macromolecules: (1) simplified schematic showing fragmentation of a macromolecule due to the collapse of cavitation microbubbles. The solvent molecules are shown as light blue spheres and the macromolecule are represented by green spheres. Subfigures (a) to (d) show the time evolution of the process: (a) ultrasound causes the appearance and implosion of transient cavitation bubbles resulting in shockwaves propagating through the solution; (b) solvent molecules compress during the pressure increase stage and decompress during the pressure decrease stage; (c) the compression and decompression of solvent molecules flowing past the macromolecule cause velocity gradients across the macromolecules; and (d) cleavage occurs when the shear stresses reach a critical value. (2) Experimental data showing the ultrasonic degradation of polystyrene (with an initial molecular weight of 411 000 g mol^−1^) in tetrahydrofuran, using an ultrasonic horn with a power of 48 W, operated at 20 kHz. (*) denotes the degree of polymerization at the at the start of sonication and (**) the degree of polymerization after 90 minutes. Adapted with permission from Smith and Temple.^261^ Copyright 1968 American Chemical Society. Created with https://Biorender.com.

Shear stresses as a result of the interaction between the polymers and the shockwaves are thought to cause molecular dissociation with subsequent breakage in the middle of the chain.^23^ Gooberman proposed a hypothesis for the underlying mechanism,^260^ shown in Fig. 8. In the same study, the shockwaves orientation was correlated with the likelihood of rupture near the polymer chain center, though it is also stated that this would require “fairly rigid linear macromolecules”.^260^ The breakage of the polymeric chain in the middle was confirmed by more studies for different polymers and solvent combinations.^254,261–263^ However, it was also reported that the possibility of multiple points of chain breakage should be considered for polymers with molecular weights in the 10^6^ range.^264^ It should be mentioned that when degradation occurs specifically in water, part of the effects could be attributed to chemical phenomena (see Section 4.1) and not solely to the mechanical effects discussed here.^265^ In the case of PMMA for example,^265^ approximately 30% of the degradation was attributed to the generation of free ˙OH and ˙HO_2_ radicals induced by sonication at 250 kHz, and power below 20 W (measured quantitatively by the extent of oxidation of KI).

As agglomeration in crystallization is usually unwanted to avoid entrapment of impurities between the primary crystals,^13,266^ ultrasound can be used to break agglomerated crystals. Guo *et al.* demonstrated that in a slurry of sugar crystals in ethanol, both interparticle collisions and particle–shockwave interactions contribute to the breakage (for 20 kHz, at 1.16 W mL^−1^).^267^ Gielen *et al.* have shown that sonication as a post-treatment technique may be a viable approach to break some weakly-bounded agglomerates.^145^ In their study, an ultrasound post-treatment (at 30 kHz with 0.15 W mL^−1^) of 60 min removed most small particles attached on the surface of an API, but larger agglomerates only exhibited surface erosion.^145^ Deagglomeration has also been reported for inorganic material synthesis. Ultrasonication (at 40 kHz and powers densities ranging from 0.8 to 5 W mL^−1^) during the synthesis of Cu nanoparticles resulted in an average diameter decrease from 520 to 167 nm and a size dispersion decrease from 94 to 44 nm.^268^ This was attributed to the ultrasound preventing the aggregation (deagglomeration) of crystallites and was modelled as a competition between surface energy and ultrasonic force.^268^ In batch reactors, sonication at 20 or 24 kHz for up to 180 min could reduce the number of agglomerates during FAU synthesis.^269^ In a 60 L flow system, an ultrasonic horn operating at 20 kHz effectively deagglomerated and homogenized the zeolite particle size distribution.^270,271^ In microscale reactors, the use of low frequency (20 or 40 kHz) ultrasonic transducers can counteract clogging and solid deposition.^246,272^

#### 5.2.3 Mechanical effects: surface and structural modification

Most surfaces, even the hardest ones, can be affected by ultrasonic post-treatment,^273^ due to the anti-symmetric jets generated by the implosion of cavitation bubbles near those surfaces^273^ (as graphically illustrated in Fig. 2B(v)). These microjets can possess sufficient energy to “treat” the surface by breaking bonds, affect its roughness (*e.g.*, for subsequent coating preparation),^274^ erode it, and functionalize it. Additionally, as discussed in Section 4.1, they can even enhance chemical reaction kinetics. Occasionally, these effects are intentional, while in other instances, undesired.

Examples of ultrasound-assisted surface treatment and structural modification include the HF removal of polyvinylidene difluoride (PVDF), for subsequent grafting.^40,275^ This process consists of the elimination of HF units and subsequent formation of C=C double bonds in the polymer backbone in presence of a base and a phase transfer catalyst.^275^ Compared to conventional stirred dehydrofluorination, sonication in an ultrasonic bath of the polymer in alkaline solution provided better control over the obtained surface layer thickness. Urban and Salazar-Rojas proposed a new mechanism for surface HF removal in presence of ultrasound: in the case of conventional dehydrofluorination, the formation of C=C increases monotonously with time and temperature, which means also an increase in the surface layer thickness.^275^ On the other hand, ultrasonic dehydrofluorination reaches a plateau in the C=C bond formation, meaning a constant surface layer thickness over time.^275^ The authors hypothesize that the energy introduced in the system through sonication serves a dual purpose: enhancing dehydrofluorination kinetics on one the hand, while also facilitating the early stages of surface decomposition on the other. These two effects balance each other, creating a controlled and constant dehydrofluorinated layer on PVDF surfaces. Moreover, sonication allowed the grafting of functional silicon phthalocyanine dichloride onto the surface of dehydrofluorinated PVDF in presence of chloroplatinic acid in aqueous environment.^276^

Similarly, aluminosilicates undergo ultrasound-assisted Si and Al removal inside their framework, to tune the Si/Al ratio and induce mesoporosity. Here, two different extraction mechanisms are possible: dealumination with acid,^277^ and desilication with basic solvent.^278^ In both cases, ultrasound, by improving the contact between the solids and the extractant, enhances the hydrolyzation of the framework, bond cleavage, and therefore Al or Si extraction.^277,278^ Shu *et al.* suggest that it is thanks to the high local temperature rise after particle–particle collisions, that the extraction agent vaporizes at the solid–liquid interface, enabling dealumination of zeolite A by thionyl chloride at room temperature instead of 300 °C.^279^ In the case of FAU desilication, the use of ultrasound reaches the same desilication levels at ambient conditions as compared to standard desilication at 80 °C.^280^ Dealumination of zeolite Y in a 28 kHz, 600 W ultrasonic bath increased the degree of dealumination (%) in all tested cases.^277^ Catalytic tests for xantane synthesis with the dealuminated zeolite showed an increase in yield from 80% to 95%, in a reaction time of 35 and 16 min for the silent and sonicated case, respectively.^277^ Ultrasonic Al extraction is capable to retain a higher crystallinity of the sample compared to standard, whereas for Si extraction in both cases the crystallinity is partially compromised. In the case of desilication, Oruji and coworkers showed that zeolite Y was able to increase mesoporosity while retaining microporosity in the framework.^278^ They attributed the loss in crystallinity of conventionally treated zeolites to the loss of the micropore structure, and of ultrasonically-treated zeolites to the formation of defects on the solids’ surface. Nevertheless, the mechanism underlying this hypothesis still remains elusive.

**Fig. 9:**
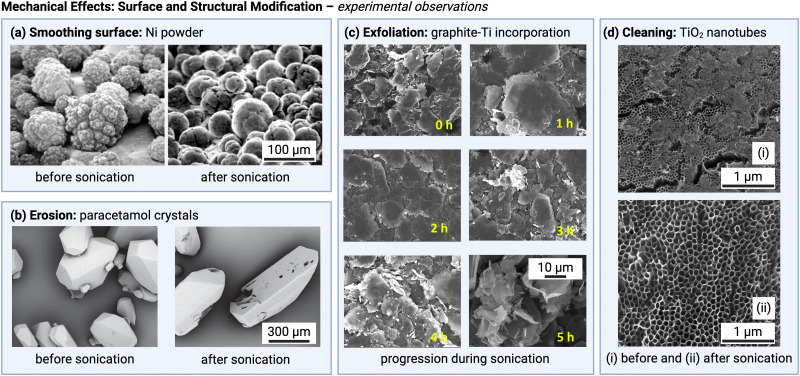
Different experimental observations related to the mechanical effect of surface and structural modification across different length scales and material classes: (a) smoothing of Ni powder particles (imaged with SEM) as a consequence of ultrasonic irradiation Adapted from Suslick and Casadonte^63^ with permission from Elsevier. (b) Example of paracetamol crystals (imaged with SEM) before and after post-treatment with ultrasound at 10 W for 30 min (0.04 W mL^−1^, at 30 kHz). Adapted from Gielen *et al.*^203^ with permission from John Wiley and Sons. (c) FESEM images of the titanium alloy particle incorporation into the exfoliated graphite due to horn corrosion by ultrasound (at 50 W over 5 h). Adapted from Karikalan *et al.*^283^ with permission from Elsevier Copyright 2019. (d) The surface of TiO_2_ nanotube arrays (imaged with SEM) before and after ultrasonic post-treatment (in an ultrasonic bath for 10 min).^284^ Ultrasonication removed nanotube fragments from the surface of the nanotubes. Adapted from Yi *et al.*,^284^ with permission from Elsevier licensed under a Creative Commons CC-BY license. Created with https://Biorender.com.

In the case of metal catalysts, post-treatment with ultrasound can enhance their catalytic performance by providing additional interface for transient cavitation to occur (see Section 5.2.4), by “activating” the solid surface as a consequence of particle–particle collisions (as discussed in Section 5.2.2), or by eroding the surface after jet impingement. The removal of the surface oxide layer, and hence metallic reduction, is proven *via* macroscopic smoothing of the particle surface, as shown in Fig. 9(a).^40^ In another study by Suslick and Casadonte otherwise inactive Ni powder was activated through the use of ultrasound (at 20 kHz and 50 W cm^−2^).^281^ After sonication for 1 h, the metal became active to hydrogenation of alkenes. Similar activities for this reaction were found when using metal Ni sponges. However, in the case of ultrasonically activated Ni powder, the observed effect could be attributed by the change in surface morphology and by the extent of aggregation due to interparticle collision. In that case, the oxide layer upon the particle was thinned by the use of ultrasound, which had a cleaning action that contributed to the activation of the catalyst. In certain metal-catalyzed reaction, the exposure of a larger active surface after sonication leads to an increased number of electrons available for enhancement of reactions based on electron transfer mechanisms.^282^

Ultrasound can also be used as a post-processing technique for material functionalization. This is usually achieved by applying different techniques, such as metal impregnation or ion exchange, where the contact between the solid material and the solvent plays a fundamental role in the process.^285^ In the case of ultrasound-assisted zeolite functionalization, improved metal cation dispersion over the supportive material is achieved, in order to enhance the products catalytic properties.^199^ Compared to conventional stirred impregnation, ultrasonic impregnation results in stronger metal oxide anchoring on the framework, which leads to a variation of the acid site type in the zeolite.^286^ This results in a more favorable zeolite-supported acid esterification process. It has also been shown that ultrasonic impregnation reduces the metal particle size achieving better active metal dispersion, which could help, for example, in the preparation of nanocatalysts.^287^ Applying ultrasound (*e.g.*, 20 kHz and 60 W for 20 min) during ion exchange can either shorten process times from one day to 20 minutes,^288,289^ or achieve complete exchange without providing external heating and lower process temperatures, whilst also improving final material performance through better metal loading as a result of more homogeneous dispersion.^290^ Ultrasonicated samples retain and may even improve their microporosity compared to samples that undergo standard ion exchange.^290^ In some cases, also metal reduction on the zeolitic support was reported.^291,292^ Here, it is hypothesized that the presence of shockwaves causes metal migration towards the surface, which is eventually also impacted by erosion. Nonetheless, it is hypothesized that radicals play a pivotal role in the metal reduction reaction. Talebi *et al.* proposed a radical reduction mechanism of Ag^−^ in the presence of alcoholic additives, but they also state that the sole presence of H radicals after water sonolysis could be exploited as a reducing agent.^292^ Similarly, the synergistic effect of enhanced transport properties and radical formation enhanced the decomposition of oxidizing agents in the case of surface hydrophilization of polymers. Here, the use of ultrasonication enabled the use of mild oxidizing agents instead of more aggressive formulations and accelerated the oxidation rate.^293–295^ These studies showed that a level of control of the oxidation process was achievable by varying the sonication time, intensity, and reaction temperature.^293,294^

Ultrasound has also been utilized to modify the structure of carbon nanotubes, which are quasi-one-dimensional nanostructures known for their desirable electronic and thermal stability properties, as well as mechanical strength.^29,296,297^ This process helps to disperse the nanotubes, by breaking up the clumps, which allows them to be more effectively utilized in various (either chlorinated or surfactant-stabilized aqueous) solvents despite their inherent insolubility.^29^ Sonication (in an ultrasonic bath at 40.5 kHz and ambient conditions) effectively dispersed single-walled carbon nanotubes in o-dichlorobenzene through sonochemical decomposition and polymerization of the solvent, which irreversibly adheres (possibly during the termination step) to the tubes’ surfaces.^297^ Moonoosawmy and Kruse studied this observation further and found that sonication affects the carbon nanotubes’ electronic band structure in the presence of Fe particles^296^ (a common catalyst during the tubes’ preparation).^29^ The use of ultrasound can however also induce structural and surface damage to carbon nanotubes, and in some cases, even cause them to fracture (break open, thereby causing a length reduction),^29^ through processes such as bending and buckling, and stretching and rupture.^49^ Sesis *et al.* showed that ultrasound-induced (at 20 kHz) stable cavitation led to sonochemical surface damage, whereas higher powers led to transient cavitation resulting in length reduction and exfoliation (see further).^48^

In the case of weakly bonded layered materials, such as graphite (with a crystal structure consisting of carbon sheets held together by weak van der Waals forces^29^), an external energy field, like ultrasound, can destroy the interlayer bonds, resulting in the formation of nanosheets in a process known as exfoliation.^283^ The exfoliated and expanded (“puffed-up”) material can then, for example, serve as a filler in composites. Field emission scanning electron microscopy (FESEM) images of graphite exfoliation are shown in Fig. 9(c). In this specific example, titanium alloy particles detached from the titanium tip during sonication (for 1 to 5 h, at 20 kHz with 0.5 W mL^−1^) and were then incorporated in the exfoliated graphite over time.^283^ This illustrates that sonication can impact both the solid being processed as well as the material through which the sound waves are delivered.^283^

In some cases the use of ultrasound is even more explicitly detrimental for the product quality, such as in the post-treatment of pharmaceutical-grade crystals. Images of paracetamol crystals, before and after sonication are shown in Fig. 9(b),^203^ indicating significant pit erosion and edge defects on the crystals surface (after sonication at 30 kHz with 0.04 W mL^−1^ for 30 min). Ultrasonic treatment is also known to induce edge defects on the crystals’ surfaces.^14,203,298^ Moreover, ultrasound-induced erosion could also result in contamination of the crystal suspension.^145^ Uncertainty arises in discussions regarding the impact of ultrasound on crystal shape. Some studies have shown that ultrasound can induce a more spherical form, often referred to as “polishing the solid” (*e.g.*, ref. 94 and 299–301), or at least render the particles more regular in shape. Continuous barium sulfate precipitation without ultrasonication produced large, flaky, flat, sharp-angled particles.^300^ However, when ultrasound was applied (at 20 kHz and 20–160 W), small, spherical, round particles were obtained.^300^ For manganese carbonate precipitation, the smallest and most spherical precipitates were obtained at a high ultrasonic intensity (49 W L^−1^ compared to 25 and 4 W L^−1^) and a low frequency of 94 kHz (compared to 577 and 1134 kHz).^250^ However, there are also reports that state that the prismatic shape of paracetamol remains unaffected for sonication at 20 kHz and 0.33 W mL^−1^, with only the surface texture being altered.^203,302^ These examples illustrate the difficulty in predicting *a priori* which mechanism (*e.g.*, erosion *versus* fragmentation) and to what extent (*e.g.*, pitting erosion *versus* polishing) will dominate under specific conditions.

#### 5.2.4 Transport effects: presence of interfacial area

The addition of solids in a solution introduces solid–liquid interfacial area, which can act as a nucleus for triggering the formation of cavitation bubbles.^30^ Bubble nucleation as described by the classical nucelation theory is dependent on the solid–liquid contact angle: bubble nucleation thus occurs preferentially on hydrophobic surfaces.^30,31^ In addition, other solid material properties play a role during sonocatalytic mechanisms, such as: particle size, surface roughness, wettability, and the presence of pores.^30,303^ Zhang *et al.* showed that the intensity of cavitational activity is highly dependent on the initial solid catalyst size and the apparent contact angle between the cavitation nucleus and the solid itself, but also bubble at its critical growth size and the catalyst.^304^ Bremond and co-workers designed micro-patterned perfluorodecyltrichlorosilane-coated (*i.e.*, hydrophobic) silicon wafers with well-defined cavities in order to quantitatively control nucleation of cavitation bubbles through a liquid phase pressure swing.^303^

Heterogeneous sonocatalysis is largely applied to enhance degradation reactions, in particular for the removal of organic pollutants in wastewater treatment.^30,140,305^ The intrinsic material properties (*e.g.*, conductivity, mechanical strength, surface area, presence of specific functional groups, porosity *etc.*) determine which type of sonocatalyst is the best to selectively attack pollutants.^30,140^ For example, carbon nanotubes, besides enhancing radical formation by increasing cavitational activity, are capable of selectively adsorbing organic pollutants from water, such as ibuprofene and sulfamethoxazole.^140,306^

## 6 Heuristics for targeted ultrasonic application

The research highlighted in this paper provides an overview of what can be expected from ultrasonic application for the synthesis and post-treatment processing of solids. Maximizing the effectiveness of ultrasound hinges on selecting the appropriate operational regime. From the preceding discussion, we propose a set of qualitative heuristics to guide targeted application.

While most commercial ultrasonic transducers typically operate at 20 or 40 kHz,^4^Fig. 3 illustrates that the optimal frequency should be selected according to the specific desired effect. It is advisable to gradually increase ultrasonic power density until adverse effects emerge, such as excessive erosion or purity issues for what concerns the final product, or temperature limitations and process stability for what concerns the system. This is particularly important near the cavitation threshold, where small adjustments are needed to account for the highly nonlinear effects of cavitation. In such cases, it could be more effective to limit the ultrasound duration to activate or fine-tune a specific part of the process without interfering with other stages and preventing the aforementioned side effects. Thus, also sonication time should be matched to the desired effect. Continuous ultrasound is most effective (and preferred) unless processing delicate (*e.g.*, temperature-sensitive) materials or if energy costs are a concern (*e.g.*,^184^). In such cases, a pulsation frequency can be chosen without majorly sacrificing on performance.^246^

Continuous ultrasonication at high power densities often severely affects the system's temperature. While this is sometimes a desired effect (*e.g.*, to reduce energy consumption from external heating, or to increase reaction kinetics), it can be difficult to control and optimize. In most cases, it is thus advised to operate at the optimal process temperature (*via* external temperature control), whilst exploiting advantages from other ultrasonic effects. Additionally, the transducer's temperature must remain below the Curie point. Similarly, while cavitation effects improve at lower pressures, it is generally advisable to conduct the process under normal pressure conditions. Solid concentration influences several effects (*e.g.*, erosion and attrition through particle–particle collisions), but it is often a parameter that cannot be chosen freely. Therefore, adjusting power density based on solid concentration is usually more practical. Lastly, also the reactor geometry should be designed to leverage the underlying ultrasonic phenomena, optimizing the system for its intended application. This review advocates for reactor customization, particularly when acoustic streaming or acoustophoresis is desired. In general, direct immersion of a transducer is preferable, though it may introduce impurities or particle–horn interactions.

## 7 Future perspectives

This review underscores the extensive efforts dedicated to understanding the impact of ultrasound on solid materials’ synthesis and processing. However, the complex relationship between specific ultrasonic phenomena – namely, cavitation, streaming, and wave propagation – and their effects on the solids formation or post-processing remains inadequately explored. Often, such relationships are inferred qualitatively from experimental outcomes without being the core topic of the study, leading to a lack of clarity and sometimes ambiguity in observations seen in ultrasonic processing.

To address these knowledge gaps effectively, this review advocates for the establishment of a qualitative ultrasonic phenomenon–mechanism–effect framework. Subsequent research should prioritize understanding observations within this framework. In addition, this review highlights the imperative for two distinct avenues of future research in the realm of ultrasonic processing for solids synthesis and processing.

The first trajectory entails an inward-looking exploration aimed at quantifying relationships outlined qualitatively in the current review. The ultimate and ambitious goal should be to obtain a quantitative ultrasound phenomenon–mechanism–effect framework which can be interpreted within the context of a specific system and setup. While this work initiates steps towards bridging this gap, further research demands a more systematic approach. This involves employing highly-controlled and well-characterized systems, in terms of ultrasonic phenomena, alongside the use of well-defined material systems. For the formation stage, for example, this implies that the physicochemical properties of reactants and solvents, and kinetic parameters of solids’ synthesis must be well-defined (*e.g.*, homogeneous nucleation). Such experiments require a lot of controls to establish that the influence is only due to ultrasound and its associated phenomena and not, *e.g.*, due to different heat transfer geometries or small nucleation differences. In zeolite synthesis, for instance, this would entail controls in the ultrasonic reactor in silent mode but with the same geometry and empty head space, opposed to silent reactions in classic batch autoclaves. For the post-processing stage, knowing the relevant solid properties is particularly useful. Ideally, specific effects can be studied in isolation such that the underlying mechanisms can be quantified more easily (in control experiments). For instance, one could quantify the shear forces produced during cavitation collapse, a challenging task on its own,^42^ along with the frequency of bubble implosions, and subsequently correlate these factors with the fragmentation behavior based on the material's strength properties. Such research will require coupling experimental investigations with computational studies. From an experimental point of view, such isolation studies necessitate dedicated research into controlling the spatial (*i.e.*, in the order of mm^307^) and temporal aspects (*i.e.*, in the order of ms^307^) of the specific ultrasonic phenomena.

Simultaneously, the second trajectory should adopt an outward-looking perspective, dedicated to uncovering novel applications based on the effects (or mechanisms) of ultrasound and to industrialize successful applications. Such an approach is more aligned with ongoing trends in the field.^47^ This entails, on one hand, the exploration of novel ultrasonic reactors geared towards expediting the industrial adaptation of ultrasonic technology, particularly in continuous processing which holds significant promise. An example of a potential large-scale application with considerable industrialization promise includes the degradation of solid pollutants from wastewater streams through heterogeneous sonocatalysis. An example of a smaller-scale application that seems promising for industrialization is the production of highly-tailored nanoparticles with ultrasound, in which ultrasonic cavitation mechanisms are exploited for dispersion and/or functionalization. On the other hand, it involves harnessing the potential of ultrasound in material processing areas that have yet to be fully explored. This review has already touched on the fact that there may still be certain material classes (*e.g.*, zeolites, MOFs, and COFs) where there is still untapped potential for ultrasound, based on similar behaviours observed in other types of solids’ processing.

By concurrently pursuing both trajectories, they mutually reinforce each other. For instance, a better mechanistic relationship between effect and observation in established systems, can provide a better prediction of how ultrasound will affect the synthesis of different solid materials. In summary, future research endeavours in ultrasonic processing for solids’ synthesis and processing should be guided by these dual trajectories, focusing on both the systematic elucidation of direct relationships between ultrasonic phenomena and effects observed during solid materials synthesis and processing, and the exploration of novel applications and advancements in ultrasonic technology across various domains.

## 8 Concluding remarks

Considerable research has been devoted to exploring the potential of ultrasound in solid materials’ synthesis and processing. The intricate relationship between ultrasonic phenomena, their effect on solids and the precise mechanisms underlying these effects is often overlooked in literature. The presented review addresses this gap by introducing a novel framework of ultrasonic mechanisms and the effects that ultrasound induces during the synthesis and processing of solids. Ultrasound effects are categorized by type (chemical, mechanical, and transport). The underlying mechanisms behind them are discussed within the context of the ultrasonic phenomena that can occur in solution. A summary of the framework is shown in Table 2.

**Table 2:** Summary of the relationship between underlying ultrasonic phenomena, mechanisms, and effects that can occur during the formation or post-processing of solids within liquids. The effects are divided based on their type: chemical, mechanical, and transport

Ultrasonic phenomenon	Mechanism	Effect	Effect type
Cavitation	Hotspot formation (collapse)	Local temperature and pressure increase	Transport
		Radical formation	Chemical
	Shockwaves (symmetric collapse)	Local fluid shear increase	Mechanical
		Fragmentation	Mechanical
		Deagglomeration	Mechanical
		Local concentration gradients	Transport
	Microjets (asymmetric collapse)	Surface erosion	Mechanical
	Presence of gas bubbles	Increased interfacial area	Transport
Streaming	Viscous attenuation	Bulk temperature increase	Transport
	Fluid movement	Mass transfer increase	Transport
Wave propagation	Viscous attenuation	Bulk temperature increase	Transport
	Fluid movement	Mass transfer increase	Transport

Ultrasound propagation in a solution induces four phenomena in a liquid solution, each varying in prevalence and importance: acoustophoresis, wave propagation, acoustic streaming, and acoustic cavitation. Chemical effects arise from the formation of radicals which occurs during the violent implosions of cavitation bubbles at low ultrasonic frequencies. Mechanical effects encompass all effects related to mechanical forces, such as shear forces during solid material synthesis, as well as fragmentation, deagglomeration, and erosion occurring during the post-treatment stage. These effects (primarily) stem from shockwaves and microjets associated with symmetric and asymmetric cavitation implosions, respectively. Shear effects can also occur at high frequencies in presence of acoustic streaming. Transport effects encompass changes in concentration, temperature, and pressure, both locally and throughout the bulk of the system. Bulk effects are (generally) associated with fluid movement induced by streaming (either cavitation or acoustic) or wave propagation, and the resulting viscous dissipation. Local effects are the result of hotspot formation during cavitation implosion. Typically numerous ultrasound phenomena, mechanisms, and effects interplay (simultaneously) during both formation and post-treatment stages which makes the isolation of single effects challenging. The proposed effect-mechanism framework assists in effectively interpreting the majority of reported effects.

In summary, the presented review provides a framework of different ultrasonic effects that occur during solids’ processing with ultrasound. A solid foundation is set for a deeper understanding between major phenomena, mechanisms and their effects across different material classes. This review paves the way for a more strategic and targeted application of ultrasound technology in solids’ synthesis and processing to achieve the desired solid product characteristics such as size, surface, shape, and composition.

## Abbreviations

US: Ultrasound
LFUS: Low-frequency ultrasound
HFUS: High-frequency ultrasound
PSD: Particle size distribution
MOF: Metal organic framework
SEM: Scanning electron microscopy
COFs: Covalent organic frameworks
PMMA: Poly(methyl methacrylate)
PVDF: Polyvinylidene difluoride
FESEM: Field emission scanning electron microscopy
DMF: Dimethylformamide
DMAc: Dimethylacetamide

## Author contributions

C. D.: conceptualization, visualization, writing – original draft; A. B.: conceptualization, visualization, writing – original draft; E. B: conceptualization, visualization, writing – original draft; G. D. S: writing – review & editing, supervision; M. D.: writing – review & editing, supervision; T. V. G.: writing – review & editing, supervision, funding acquisition; S. K.: writing – review & editing, supervision, funding acquisition. The manuscript was written through contributions of all authors. All authors have given approval to the final version of the manuscript.

## Conflicts of interest

There are no conflicts to declare.

## Data Availability

No primary research results, software or code have been included and no new data were generated or analysed as part of this review.

## References

[cit1] Price G. J. (2009). Polym. Int..

[cit2] Wang B., Wu J., Yuan Z. Y., Li N., Xiang S. (2008). Ultrason. Sonochem..

[cit3] Cravotto G., Cintas P. (2006). Chem. Soc. Rev..

[cit4] Meroni D., Djellabi R., Ashokkumar M., Bianchi C. L., Boffito D. C. (2022). Chem. Rev..

[cit5] Van Gerven T., Stankiewicz A. (2009). Ind. Eng. Chem. Res..

[cit6] Hem S. L. (1967). Ultrasonics.

[cit7] Ruecroft G., Hipkiss D., Ly T., Maxted N., Cains P. W. (2005). Org. Process Res. Dev..

[cit8] McCausland L. J., Cains P. W. (2004). Biotechnol. Genet. Eng. Rev..

[cit9] Jordens J., Gielen B., Xiouras C., Hussain M. N., Stefanidis G. D., Thomassen L. C., Braeken L., Van Gerven T. (2019). Chem. Eng. Process..

[cit10] Banakar V. V., Sabnis S. S., Gogate P. R., Raha A., Saurabh (2022). Chem. Eng. Res. Des..

[cit11] Sander J. R., Zeiger B. W., Suslick K. S. (2014). Ultrason. Sonochem..

[cit12] Luque de Castro M. D., Priego-Capote F. (2007). Ultrason. Sonochem..

[cit13] Nalesso S., Bussemaker M. J., Sear R. P., Hodnett M., Lee J. (2019). Ultrason. Sonochem..

[cit14] Ratsimba B., Biscans B., Delmas H., Jenck J. (1999). KONA Powder Part. J.

[cit15] Xiouras C., Fytopoulos A., Jordens J., Boudouvis A. G., Van Gerven T., Stefanidis G. D. (2018). Ultrason. Sonochem..

[cit16] Zhang Z., Sun D.-W., Zhu Z., Cheng L. (2015). Compr. Rev. Food Sci. Food Saf..

[cit17] Zamanipoor M. H., Mancera R. L. (2014). Trends Food Sci. Technol..

[cit18] Deora N. S., Misra N. N., Deswal A., Mishra H. N., Cullen P. J., Tiwari B. K. (2013). Food Eng. Rev..

[cit19] Suslick K. S., Casadonte D. J., Green M. L., Thompson M. E. (1987). Ultrasonics.

[cit20] McKenzie T. G., Karimi F., Ashokkumar M., Qiao G. G. (2019). Chem. – Eur. J..

[cit21] Kumar A. R. S. S., Padmakumar A., Kalita U., Samanta S., Baral A., Singha N. K., Ashokkumar M., Qiao G. G. (2023). Prog. Mater. Sci..

[cit22] Price G., West P., Smith P. (1994). Ultrason. Sonochem..

[cit23] Basedow A. M., Klaus H. E. (1977). Adv. Polym. Sci..

[cit24] Hinman J. J., Suslick K. S. (2017). Top. Curr. Chem..

[cit25] Gedanken A. (2004). Ultrason. Sonochem..

[cit26] Xu H., Zeiger B. W., Suslick K. S. (2013). Chem. Soc. Rev..

[cit27] Cravotto G., Gaudino E. C., Cintas P. (2013). Chem. Soc. Rev..

[cit28] Safarifard V., Morsali A. (2015). Coord. Chem. Rev..

[cit29] Skrabalak S. E. (2009). Phys. Chem. Chem. Phys..

[cit30] Qiu P., Park B., Choi J., Thokchom B., Pandit A. B., Khim J. (2018). Ultrason. Sonochem..

[cit31] Shchukin D. G., Skorb E., Belova V., Möhwald H. (2011). Adv. Mater..

[cit32] Askari S., Miar Alipour S., Halladj R., Davood Abadi Farahani M. H. (2013). J. Porous Mater..

[cit33] Mumtaz F., Irfan M. F., Usman M. R. (2021). J. Iran. Chem. Soc..

[cit34] Deneyer A., Ke Q., Devos J., Dusselier M. (2020). Chem. Mater..

[cit35] Askari S., Bashardoust Siahmard A., Halladj R., Miar Alipour S. (2016). Powder Technol..

[cit36] Vaitsis C., Sourkouni G., Argirusis C. (2019). Ultrason. Sonochem..

[cit37] Koo S., Kang D. W. (2023). CrystEngComm.

[cit38] Athanassiadis A. G., Ma Z., Moreno-Gomez N., Melde K., Choi E., Goyal R., Fischer P. (2022). Chem. Rev..

[cit39] Peters D. (1996). J. Mater. Chem..

[cit40] Suslick K. S., Price G. J. (1999). Annu. Rev. Mater. Sci..

[cit41] Eskin D., Komarov S., Tzanakis I. (2023). Ultrason. Sonochem..

[cit42] Mondal J., Lakkaraju R., Ghosh P., Ashokkumar M. (2021). Biophys. Rev..

[cit43] Mason T. J. and Vinatoru M., Sonochemistry: Fundamentals and Evolution, De Gruyter, Berlin, Boston, 2023

[cit44] Mason T. J. and Vinatoru M., Sonochemistry: Applications and Developments, De Gruyter, Berlin, Boston, 2023

[cit45] Gogate P. R. , in Theory of Cavitation and Design Aspects of Cavitational Reactors, ed. M. Ashokkumar, Springer, Netherlands, Dordrecht, 2011, pp. 31–67

[cit46] Tobergte D. R. and Curtis S., Ultrasound Technologies for Food and Bioprocessing, Springer, New York, New York, NY, 2011, vol. 53, pp. 1689–1699

[cit47] Manickam S., Camilla Boffito D., Flores E. M., Leveque J.-M., Pflieger R., Pollet B. G., Ashokkumar M. (2023). Ultrason. Sonochem..

[cit48] Sesis A., Hodnett M., Memoli G., Wain A. J., Jurewicz I., Dalton A. B., Carey J. D., Hinds G. (2013). J. Phys. Chem. B.

[cit49] Rennhofer H., Zanghellini B. (2021). Nanomaterials.

[cit50] Mat-Shayuti M. S., Tuan Ya T. M. Y. S., Abdullah M. Z., Megat Khamaruddin P. N. F., Othman N. H. (2019). Environ. Sci. Pollut. Res..

[cit51] McDonnell C. K., Lyng J. G., Arimi J. M., Allen P. (2014). Innovative Food Sci. Emerging Technol..

[cit52] Su J., Cavaco-Paulo A. (2021). Ultrason. Sonochem..

[cit53] O’Sullivan J., Murray B., Flynn C., Norton I. (2016). Food Hydrocolloids.

[cit54] Dalecki D. (2004). Annu. Rev. Biomed. Eng..

[cit55] Izadifar Z., Babyn P., Chapman D. (2017). Ultrasound. Med. Biol..

[cit56] Cai B., Mazahreh J., Ma Q., Wang F., Hu X. (2022). Int. J. Biol. Macromol..

[cit57] Richards W. T., Loomis A. L. (1927). J. Am. Chem. Soc..

[cit58] Szent-Gyorgyi A. (1933). Nature.

[cit59] Kortnev A., Martynovskaya N. (1974). Sb., Mosk. Inst. Stali. Splavov.

[cit60] Klink A. , Midler M. and Allegretti J., Chemical Engineering Progress Symposium Series, 1971

[cit61] Polotskii I. and Levin G., Sbornik Nauchnykli Robot Inslilula Melallofiziki Akademiya Nauk, USSR, 1961, vol. 13, p. 24

[cit62] Weissler A. (1953). J. Acoust. Soc. Am..

[cit63] Suslick K. S., Casadonte D. J., Doktycz S. J. (1989). Solid State Ionics.

[cit64] Inui T., Kang M. (1997). Appl. Catal., A..

[cit65] Lu K., Lago R., Chen Y., Green M., Harris P., Tsang S. (1996). Carbon.

[cit66] Nerom M. V., Gelin P., Hashemiesfahan M., Malsche W. D., Lutsko J. F., Maes D., Galand Q. (2022). Crystals.

[cit67] Devos C., Doyle A., Van Eersel M., Van Gerven T., Kuhn S. (2024). Ind. Eng. Chem. Res..

[cit68] Dong Z., Udepurkar A. P., Kuhn S. (2020). Ultrason. Sonochem..

[cit69] Certain data included herein are derived from Clarivate InCites. Copyright Clarivate 2024. All rights reserved

[cit70] Yoo J. T., Lee S. H., Hirata S., Kim C. R., Lee C. K., Shiraki T., Nakashima N., Shim J. K. (2015). Chem. Lett..

[cit71] Yang S. T., Kim J., Cho H. Y., Kim S., Ahn W. S. (2012). RSC Adv..

[cit72] Oliveira C. J., Freitas S. K., de Sousa I. G. P., Esteves P. M., Simao R. A. (2020). Colloids Surf., A..

[cit73] Li X., Yang C., Sun B., Cai S., Chen Z., Lv Y., Zhang J., Liu Y. (2020). J. Mater. Chem. A.

[cit74] Zhao W., Yan P., Li B., Bahri M., Liu L., Zhou X., Clowes R., Browning N. D., Wu Y., Ward J. W., Cooper A. I. (2022). J. Am. Chem. Soc..

[cit75] Zhao W., Yan P., Yang H., Bahri M., James A. M., Chen H., Liu L., Li B., Pang Z., Clowes R., Browning N. D., Ward J. W., Wu Y., Cooper A. I. (2022). Nat. Synth..

[cit76] Legay M., Gondrexon N., Le Person S., Boldo P., Bontemps A. (2011). Int. J. Chem. Eng..

[cit77] Dong Z., Delacour C., Carogher K. M., Udepurkar A. P., Kuhn S. (2020). Materials.

[cit78] Ashokkumar M. , Ultrasonic Synthesis of Functional Materials, Springer, 2016, pp. 17–25

[cit79] Fernandez Rivas D., Kuhn S. (2016). Top. Curr. Chem..

[cit80] Mason T. J. and Lorimer J. P., Applied Sonochemistry: The Uses of Power Ultrasound in Chemistry and Processing, Wiley-VCH, 2002

[cit81] Sutkar V. S., Gogate P. R. (2009). Chem. Eng. J..

[cit82] Sancheti S. V., Gogate P. R. (2017). Ultrason. Sonochem..

[cit83] Laugier P. and Haïat G., in Introduction to the Physics of Ultrasound, ed. P. Laugier and G. Haïat, Springer, Netherlands, Dordrecht, 2011, pp. 29–45

[cit84] Louisnard O. (2012). Ultrason. Sonochem..

[cit85] Riesz P., Berdahl D., Christman C. L. (1985). Environ. Health Perspect..

[cit86] Rose J. L. , Ultrasonic Guided Waves in Solid Media, Cambridge University Press, 2014

[cit87] Pankaj and Ashokkumar M., Theoretical and Experimental Sonochemistry Involving Inorganic Systems, Springer, Netherlands, Dordrecht, 2011

[cit88] Young F. R. , Cavitation, Imperial College Press (distr. by World Scientific Publishing co.), 1999

[cit89] Jordens J., Gielen B., Braeken L., Van Gerven T. (2014). Chem. Eng. Process..

[cit90] Raso J., Mañas P., Pagán R., Sala F. J. (1999). Ultrason. Sonochem..

[cit91] Skauen D. (1976). Ultrasonics.

[cit92] Ashokkumar M. (2011). Ultrason. Sonochem..

[cit93] Yasui K. , Fundamentals of Acoustic Cavitation and Sonochemistry, Springer, Netherlands, 2011, pp. 1–29

[cit94] Jordens J., Appermont T., Gielen B., Van Gerven T., Braeken L. (2016). Cryst. Growth Des..

[cit95] Suslick K. S. (1989). Sci. Am..

[cit96] Suslick K. S., Didenko Y., Fang M. M., Hyeon T., Kolbeck K. J., McNamara W. B., Mdleleni M. M., Wong M. (1999). Philos. Trans. R. Soc., A..

[cit97] Noltingk B. E., Neppiras E. A. (1950). Proc. Phys. Soc. Sect. B.

[cit98] Doktycz S. J., Suslick K. S. (1990). Science.

[cit99] Preece C. M., Brunton J. H. (1980). Wear.

[cit100] Pecha R., Gompf B. (2000). Phys. Rev. Lett..

[cit101] Maris H. J. (2006). C. R. Phys..

[cit102] Brennen C. , Cavitation and Bubble Dynamics, Cambridge University Press, 2014

[cit103] Yasui K. , Acoustic Cavitation and Bubble Dynamics, Springer, 2018

[cit104] Leighton T. , The Acoustic Bubble, Academic Press, 1994, pp. 67–128

[cit105] Hilgenfeldt S., Brenner M. P., Grossman S., Lohse D. (1998). J. Fluid Mech..

[cit106] Bampouli A., Goris Q., Van Olmen J., Solmaz S., Noorul Hussain M., Stefanidis G. D., Van Gerven T. (2023). Ultrason. Sonochem..

[cit107] Ting R. Y. (1976). Nature.

[cit108] Bampouli A., Goris Q., Hussain M. N., Louisnard O., Stefanidis G. D., Van Gerven T. (2024). Chem. Eng. Process..

[cit109] Lesnik S., Aghelmaleki A., Mettin R., Brenner G. (2022). Ultrason. Sonochem..

[cit110] Nalajala V. S., Moholkar V. S. (2011). Ultrason. Sonochem..

[cit111] Wu J. (2018). Fluids.

[cit112] Wiklund M., Green R., Ohlin M. (2012). Lab Chip.

[cit113] Landau L. and Lifshitz E., Fluid Mechanics: Volume 6, Elsevier Science, 2013

[cit114] Lebon G. B., Tzanakis I., Pericleous K., Eskin D., Grant P. S. (2019). Ultrason. Sonochem..

[cit115] Bruus H. (2012). Lab Chip.

[cit116] Maisto A., Bilgen M., de Hemptinne A., Gelin P., Briet M., Mertens R., Gielen B., Collas A., De Malsche W. (2024). Chem. Eng. Process..

[cit117] Dong Z., Fernandez Rivas D., Kuhn S. (2019). Lab Chip.

[cit118] Lenshof A., Magnusson C., Laurell T. (2012). Lab Chip.

[cit119] Bruus H. (2011). Lab Chip.

[cit120] Bruus H. (2012). Lab Chip.

[cit121] Dual J., Schwarz T. (2012). Lab Chip.

[cit122] Dual J., Möller D. (2012). Lab Chip.

[cit123] Lenshof A., Evander M., Laurell T., Nilsson J. (2012). Lab Chip.

[cit124] Dual J., Hahn P., Leibacher I., Möller D., Schwarz T. (2012). Lab Chip.

[cit125] Glynne-Jones P., Boltryk R. J., Hill M. (2012). Lab Chip.

[cit126] Bruus H. (2012). Lab Chip.

[cit127] Glynne-Jones P., Boltryk R. J., Hill M. (2012). Lab Chip.

[cit128] Rayaroth M. P., Aravind U. K., Aravindakumar C. T. (2016). Environ. Chem. Lett..

[cit129] Mason T. J. (1997). Chem. Soc. Rev..

[cit130] Hagenson L. C., Doraiswamy L. K. (1998). Chem. Eng. Sci..

[cit131] Adewuyi Y. G. (2001). Ind. Eng. Chem. Res..

[cit132] Luche J.-L. , Synthetic organic sonochemistry, Springer Science + Business Media, New York, 1998

[cit133] Suslick K. S., Gawienowski J. J. (1984). Solutions.

[cit134] Misik V., Riesz P. (1994). J. Phys. Chem..

[cit135] Vinatoru M., Mason T. J. (2019). Ultrason. Sonochem..

[cit136] Luche J., Einhorn C., Einhorn J., Sinisterra-Gago J. (1990). Tetrahedron Lett..

[cit137] Luche J.-L. (1994). Ultrason. Sonochem..

[cit138] Cravotto G., Cintas P. (2007). Angew. Chem., Int. Ed..

[cit139] Hickenboth C. R., Moore J. S., White S. R., Sottos N. R., Baudry J., Wilson S. R. (2007). Nature.

[cit140] Gholami P., Khataee A., Soltani R. D. C., Bhatnagar A. (2019). Ultrason. Sonochem..

[cit141] Suslick K. S., Schubert P. F., Goodale J. W. (1981). J. Am. Chem. Soc..

[cit142] Ferrera-Escudero S., Perozo-Rondón E., Calvino-Casilda V., Casal B., Martín-Aranda R. M., López-Peinado A. J., Durán-Valle C. J. (2010). Appl. Catal., A.

[cit143] Kusters K. A., Pratsinis S. E., Thoma S. G., Smith D. M. (1994). Powder Technol..

[cit144] Xiouras C., Fytopoulos A. A., Ter Horst J. H., Boudouvis A. G., Van Gerven T., Stefanidis G. D. (2018). Cryst. Growth Des..

[cit145] Gielen B., Jordens J., Thomassen L. C., Braeken L., Van Gerven T. (2017). Crystals.

[cit146] Kim H. N., Suslick K. S. (2017). Chem. – Eur. J..

[cit147] Leonelli C., Mason T. J. (2010). Chem. Eng. Process..

[cit148] Garrett S. L. , Understanding Acoustics: An experimentalist's view of sound and vibration, Springer, 2nd edn, 2020 10.1121/10.0006210 34598645

[cit149] Gevari M. T., Abbasiasl T., Niazi S., Ghorbani M., Koşar A. (2020). Appl. Therm. Eng..

[cit150] Harzali H., Baillon F., Louisnard O., Espitalier F., Mgaidi A. (2011). Ultrason. Sonochem..

[cit151] Dodds J., Espitalier F., Louisnard O., Grossier R., David R., Hassoun M., Baillon F., Gatumel C., Lyczko N. (2007). Part. Part. Syst. Charact..

[cit152] Louisnard O., Gomez F. J., Grossier R. (2007). J. Fluid Mech..

[cit153] Grossier R., Louisnard O., Vargas Y. (2007). Ultrason. Sonochem..

[cit154] Kiani H., Zhang Z., Delgado A., Sun D.-W. (2011). Food Res. Int..

[cit155] Devos C., Van Gerven T., Kuhn S. (2021). CrystEngComm.

[cit156] Wohlgemuth K., Kordylla A., Ruether F., Schembecker G. (2009). Chem. Eng. Sci..

[cit157] Fatemi N., Dong Z., Van Gerven T., Kuhn S. (2019). Langmuir.

[cit158] Devos C., Brozzi E., Van Gerven T., Kuhn S. (2023). Org. Process Res. Dev..

[cit159] Yin X., Long Z., Wang C., Li Z., Zhao M., Yang S. (2019). Ultrason. Sonochem..

[cit160] van Iersel M. M., Benes N. E., Keurentjes J. T. (2008). Ultrason. Sonochem..

[cit161] Fried J. R. , Polymer Science & Technology, Pearson Education, 2014

[cit162] Billmeyer Fred W. , Textbook of Polymer Science, John Wiley & Sons, 3rd edn, 1984, vol. 12, p. 608

[cit163] Rodriguez F. , Cohen C., Ober C. and Archer L., Principles of Polymer Systems, CRC Press, 2014

[cit164] Price G. J., Norris D. J., West P. J. (1992). Macromolecules.

[cit165] Lindstrom O., Lamm O. (1951). J. Phys. Chem..

[cit166] Fujiwara H., Goto K. (1991). Polym. Bull..

[cit167] McKenzie T. G., Colombo E., Fu Q., Ashokkumar M., Qiao G. G. (2017). Angew. Chem., Int. Ed..

[cit168] Collins J., McKenzie T. G., Nothling M. D., Allison-Logan S., Ashokkumar M., Qiao G. G. (2019). Macromolecules.

[cit169] Sen M., Bose A., Pal P., Das J. K., Das N. (2014). J. Am. Ceram. Soc..

[cit170] Bose A., Sen M., Das J. K., Das N. (2014). RSC Adv..

[cit171] Pal P., Das J. K., Das N., Bandyopadhyay S. (2013). Ultrason. Sonochem..

[cit172] Feng G., Cheng P., Yan W., Borona M., Li X., Su J. H., Wang J., Li Y., Corma A., Xu R., Yu J. (2016). Science.

[cit173] Jianyu W., Qiang Z., Wenfu Y., Jihong Y. (2021). Chem. J. Chin. Univ..

[cit174] Novita T. H., Kadja G. T. M. (2024). J. Porous Mater..

[cit175] Chen X., Qiu M., Li S., Yang C., Shi L., Zhou S., Yu G., Ge L., Yu X., Liu Z., Sun N., Zhang K., Wang H., Wang M., Zhong L., Sun Y. (2020). Angew. Chem., Int. Ed..

[cit176] Wang J., Liu P., Boronat M., Ferri P., Xu Z., Liu P., Shen B., Wang Z., Yu J. (2020). Angew. Chem., Int. Ed..

[cit177] Feng G., Wang J., Boronat M., Li Y., Su J.-H., Huang J., Ma Y., Yu J. (2018). J. Am. Chem. Soc..

[cit178] Dewes R. M., Ramirez H., Valdez M., Pereira L., Gerven T. V. (2022). Ultrason. Sonochem..

[cit179] Trinh T. T., Rozanska X., Delbecq F., Sautet P. (2012). Phys. Chem. Chem. Phys..

[cit180] Sharma A., Gogate P. R. (2020). Chem. Eng. Process..

[cit181] Isariebel Q. P., Carine J. L., Ulises-Javier J. H., Anne-Marie W., Henri D. (2009). Ultrason. Sonochem..

[cit182] Tronson R., Ashokkumar M., Grieser F. (2002). J. Phys. Chem. B.

[cit183] Singla R., Grieser F., Ashokkumar M. (2009). Ultrason. Sonochem..

[cit184] Paradkar A., Maheshwari M., Kamble R., Grimsey I., York P. (2006). Pharm. Res..

[cit185] Serna-Galvis E. A., Porras J., Torres-Palma R. A. (2022). Ultrason. Sonochem..

[cit186] Forsyth C., Mulheran P. A., Forsyth C., Haw M. D., Burns I. S., Sefcik J. (2015). Cryst. Growth Des..

[cit187] Liu J., Rasmuson A. C. (2013). Cryst. Growth Des..

[cit188] Devos C., Vananroye A., Cardinaels R., Xiouras C., Van Gerven T., Kuhn S. (2023). Soft Matter.

[cit189] Devos C., Xiouras C., Van Gerven T., Kuhn S. (2024). Cryst. Growth Des..

[cit190] Myerson A. S. , Erdemir D. and Lee A. Y., Handbook of industrial crystallization, Cambridge University Press, 3rd edn, 2019, pp. 1–528

[cit191] Ramisetty K. A., Pandit A. B., Gogate P. R. (2013). Ind. Eng. Chem. Res..

[cit192] Lyczko N., Espitalier F., Louisnard O., Schwartzentruber J. (2002). Chem. Eng. J..

[cit193] Hussain M. N., Jordens J., Kuhn S., Braeken L., Van Gerven T. (2020). Chem. Eng. J..

[cit194] Gracin S., Uusi-Penttilä M., Rasmuson Å. C. (2005). Cryst. Growth Des..

[cit195] Andaç Ö., Tatler M., Sirkecioğlu A., Ece I., Erdem-Şenatalar A. (2005). Microporous Mesoporous Mater..

[cit196] Askari S., Halladj R. (2012). Ultrason. Sonochem..

[cit197] Chen C. T., Iyoki K., Yonezawa Y., Okubo T., Wakihara T. (2020). J. Phys. Chem. C.

[cit198] Olszówka J. E., Pashkova V., Kornas A., Dedecek J., Brus J., Urbanova M., Tabor E., Klein P., Brabec L., Mlekodaj K. (2021). New J. Chem..

[cit199] Kornas A., Olszówka J. E., Urbanova M., Brabec L., Rathousky J., Dedecek J., Pashkova V. (2021). ACS Omega.

[cit200] Price G. J., Hearn M. P., Wallace E. N., Patel A. M. (1996). Polymer.

[cit201] Gatumel C., Espitalier F., Schwartzentruber J., Biscans B., Wilhelm A. M. (1998). KONA Powder Part. J.

[cit202] Devos C., Van Gerven T., Kuhn S. (2021). Cryst. Growth Des..

[cit203] Gielen B., Claes T., Janssens J., Jordens J., Thomassen L. C., Gerven T. V., Braeken L. (2017). Chem. Eng. Technol..

[cit204] Jordens J., Canini E., Gielen B., Van Gerven T., Braeken L. (2017). Crystals.

[cit205] Briuglia M. L., Sefcik J., ter Horst J. H. (2019). Cryst. Growth Des..

[cit206] Kadam S. S., Kramer H. J. M., ter Horst J. H. (2011). Cryst. Growth Des..

[cit207] Xiouras C., Van Aeken J., Panis J., Ter Horst J. H., Van Gerven T., Stefanidis G. D. (2015). Cryst. Growth Des..

[cit208] Rougeot C., Guillen F., Plaquevent J.-C., Coquerel G. (2015). Cryst. Growth Des..

[cit209] Song Y., Chen W., Chen X. (2008). Cryst. Growth Des..

[cit210] Viedma C. (2005). Phys. Rev. Lett..

[cit211] Noorduin W. L., van Enckevort W. J. P., Meekes H., Kaptein B., Kellogg R. M., Tully J. C., McBride J. M., Vlieg E. (2010). Angew. Chem., Int. Ed..

[cit212] Kruus P., Patraboy T. J. (1985). J. Phys. Chem..

[cit213] Koda S., Suzuki A., Nomura H. (1995). Polym. J..

[cit214] Narmon A. S. , Jenisch L. M., Pitet L. M. and Dusselier M., Ring-Opening Polymerization Strategies for Degradable Polyesters, John Wiley & Sons, Ltd, 2022, ch. 7, pp. 205–271

[cit215] Stoessel S. J. (1993). J. Appl. Polym. Sci..

[cit216] Price G. J., Lenz E. J., Ansell C. W. (2002). Eur. Polym. J..

[cit217] Price G. J., Lenz E. J., Ansell C. W. (2002). Eur. Polym. J..

[cit218] Thompson L. H., Doraiswamy L. K. (2000). Chem. Eng. Sci..

[cit219] Haque E., Khan N., Park J., Jhung S. H. (2010). Chem. – Eur. J..

[cit220] Israr F., Chun D., Kim Y., Kim D. K. (2016). Ultrason.
Sonochem..

[cit221] Hickling R. (1965). Nature.

[cit222] Cherepanov P. V., Kollath A., Andreeva D. V. (2015). Ultrason. Sonochem..

[cit223] Reinoso D., Adrover M., Pedernera M. (2018). Ultrason. Sonochem..

[cit224] Mu Y., Zhang Y., Fan J., Guo C. (2017). Ultrason. Sonochem..

[cit225] Yit T., Ng S., Leng T., Fong Y. (2019). Mater. Today: Proc..

[cit226] Wang Y., Li X., Gao Y., Chen F., Liu Z., An J., Xie S., Xu L., Zhu X. (2021). Inorg. Chem. Front..

[cit227] Price C. (1997). Chem. Eng. Prog..

[cit228] Kruus P., Lawrie J. A., O’Neill M. L. (1988). Ultrasonics.

[cit229] Kruus P., O’Neill M., Robertson D. (1990). Ultrasonics.

[cit230] Cogné C., Labouret S., Peczalski R., Louisnard O., Baillon F., Espitalier F. (2016). Ultrason. Sonochem..

[cit231] Capellades G., Kiil S., Dam-Johansen K., Mealy M. J., Christensen T. V., Myerson A. S. (2017). Cryst. Growth Des..

[cit232] Wu J., Wang B., Li N., Xiang S. (2006). Chin. J. Catal..

[cit233] Chen Y., Truong V. N., Bu X., Xie G. (2020). Ultrason. Sonochem..

[cit234] Devarakonda S., Evans J. M. B., Myerson A. S. (2003). Cryst. Growth Des..

[cit235] Nakamura E., Imanishi Y., Machii D. (1994). J. Org. Chem..

[cit236] Ando T., Fujita M., Kimura T., Leveque J. M., Luche J. L., Sohmiya H. (1996). Ultrason. Sonochem.

[cit237] Ando T., Sumi S., Kawate T., Née Yamawaki J. I., Hanafusa T. (1984). J. Chem. Soc., Chem. Commun..

[cit238] Enomoto N. and Okitsu K., Sonochemistry and the Acoustic Bubble, Elsevier Inc., 2015, pp. 187–206

[cit239] Enomoto N., Akagi J.-i, Nakagawa Z.-e (1996). Ultrason. Sonochem..

[cit240] Ando T., Kawate T., Yamawaki) J. I. N., Hanafusa T. (2006). Chem. Lett..

[cit241] Ando T., Kimura T. (1990). Ultrasonics.

[cit242] Vinatoru M., Mason T. J. (2021). Molecules.

[cit243] Prozorov T., Prozorov R., Suslick K. S. (2004). J. Am. Chem. Soc..

[cit244] Raman V., Abbas A. (2008). Ultrason. Sonochem..

[cit245] Zeiger B. W., Suslick K. (2011). J. Am. Chem. Soc..

[cit246] Delacour C., Lutz C., Kuhn S. (2019). Ultrason. Sonochem..

[cit247] Skorb E. V., Möhwald H. (2016). Ultrason. Sonochem..

[cit248] Gao J. X., Huang H. Q., Li X. X., Du F. H., Li L. B., Li J. S. (2015). Chem. Eng. Technol..

[cit249] Lee J., Ashokkumar M., Kentish S. E. (2014). Ultrason. Sonochem..

[cit250] Jordens J., De Coker N., Gielen B., Van Gerven T., Braeken L. (2015). Ultrason. Sonochem..

[cit251] Falconer D., Liu L. X., Parrish B., Mukhtar M., Girard K. P., Thorat A. A., Marziano I., Lee J. (2024). Cryst. Growth Des..

[cit252] Narducci O. , PhD thesis, University College London Dep. of Chemical Engineering, 2012

[cit253] Thoma S. G., Ciftcioglu M., Smith D. M. (1991). Powder Technol..

[cit254] Basedow A. M., Ebert K. H. (1975). Die Makromol. Chem..

[cit255] Weissler A. (1950). J. Appl. Phys..

[cit256] Melville H. W., Murray A. J. (1950). Trans. Faraday Soc..

[cit257] Mostafa M. A. K. (1958). J. Polym. Sci..

[cit258] Mostafa M. A. K. (1958). J. Polym. Sci..

[cit259] Mostafa M. A. K. (1958). J. Polym. Sci..

[cit260] Gooberman G. (1960). J. Polym. Sci..

[cit261] Smith W. B., Temple H. W. (1968). J. Phys. Chem..

[cit262] Price G. J., Smith P. F. (1991). Polym. Int..

[cit263] Price G. J., Smith P. F. (1993). Polymer.

[cit264] Taghizadeh M. T., Bahadori A. (2009). J. Polym. Res..

[cit265] Alexander P., Fox M. (1954). J. Polym. Sci..

[cit266] Capellades G., Bonsu J. O., Myerson A. S. (2022). CrystEngComm.

[cit267] Guo Z., Jones A., Li N., Germana S. (2007). Powder Technol..

[cit268] Yang G., Lin W., Lai H., Tong J., Lei J., Yuan M., Zhang Y., Cui C. (2021). Ultrason. Sonochem..

[cit269] Ramirez Mendoza H., Jordens J., Valdez Lancinha Pereira M., Lutz C., Van Gerven T. (2020). Ultrason. Sonochem..

[cit270] Ramirez Mendoza H. , PhD thesis, KU Leuven Dep. of Chemical Engineering, 2020

[cit271] Ramirez Mendoza H., Valdez Lancinha Pereira M., Van Gerven T., Lutz C. (2020). J. Adv. Manuf. Process..

[cit272] Delacour C., Stephens D. S., Lutz C., Mettin R., Kuhn S. (2020). Org. Process Res. Dev..

[cit273] Pflieger R. , Nikitenko S. I., Cairós C. and Mettin R., Characterization of Cavitation Bubbles and Sonoluminescence, Springer, 2019

[cit274] Wolloch L., Kost J. (2010). J. Controlled Release.

[cit275] Urban M. W., Salazar-Rojas E. M. (1988). Macromolecules.

[cit276] Exsted B. J., Urban M. W. (1993). J. Inorg. Organomet. Polym..

[cit277] Hosseini M., Zanjanchi M. A., Golmojdeh H. (2015). J. Chem. Sci..

[cit278] Oruji S., Khoshbin R., Karimzadeh R. (2018). Fuel Process. Technol..

[cit279] Shu S., Husain S., Koros W. J. (2007). Ind. Eng. Chem. Res..

[cit280] Kuterasiński Ł., Rojek W., Gackowski M., Zimowska M., Jodłowski P. J. (2020). Ultrason. Sonochem.

[cit281] Suslick K. S., Casadonte D. J. (1987). J. Am. Chem. Soc..

[cit282] Taghizadeh M. T., Seifi-aghjekohal P. (2015). Ultrason. Sonochem..

[cit283] Karikalan N., Elavarasan M., Yang T. C. (2019). Ultrason. Sonochem..

[cit284] Yi Z., Zeng Y., Wu H., Chen X., Fan Y., Yang H., Tang Y., Yi Y., Wang J., Wu P. (2019). Results Phys..

[cit285] Kinger G., Lugstein A., Swagera R., Ebel M., Jentys A., Vinek H. (2000). Microporous Mesoporous Mater..

[cit286] Ketzer F., Celante D., de Castilhos F. (2020). Microporous Mesoporous Mater..

[cit287] Vafaeian Y., Haghighi M., Aghamohammadi S. (2013). Energy Convers. Manag.

[cit288] Jodłowski P. J., Czekaj I., Stachurska P., Kuterasiński Ł., Chmielarz L., Jȩdrzejczyk R. J., Jeleń P., Sitarz M., Górecka S., Mazur M., Kurzydym I. (2021). Catalysts.

[cit289] Jodłowski P. J., Kuterasiński Ł., Jȩdrzejczyk R. J., Chlebda D., Gancarczyk A., Basa̧g S., Chmielarz L. (2017). Catalysts.

[cit290] Woo J. M., Seo J. Y., Kim H., Lee D. H., Park Y. C., Yi C. K., Park Y. S., Moon J. H. (2018). Ultrason. Sonochem..

[cit291] Tanabe S., Matsumoto H., Mizushima T., Okitsu K., Maeda Y. (1996). Chem. Lett..

[cit292] Talebi J., Halladj R., Askari S. (2010). J. Mater. Sci..

[cit293] Price G. J., Keen F., Clifton A. A. (1996). Macromolecules.

[cit294] Price G. J., Clifton A. A., Keen F. (1996). Polymer.

[cit295] Cobley A. (2009). Surf. Eng..

[cit296] Moonoosawmy K. R., Kruse P. (2008). J. Am. Chem. Soc..

[cit297] Niyogi S., Hamon M. A., Perea D. E., Kang C. B., Zhao B., Pal S. K., Wyant A. E., Itkis M. E., Haddon R. C. (2003). J. Phys. Chem. B.

[cit298] Amara N., Ratsimba B., Wilhelm A. M., Delmas H. (2001). Ultrason. Sonochem..

[cit299] Jordens J., De Coker N., Gielen B., Van Gerven T., Braeken L. (2015). Ultrason. Sonochem..

[cit300] Pohl B., Jamshidi R., Brenner G., Peuker U. (2012). Chem. Eng. Sci..

[cit301] Byrne T. and Dahal M., Method of production for transition metal compound particles, Australian Pat., AU2011350062B2, 2012

[cit302] Gielen B., Kusters P., Jordens J., Thomassen L. C., Van Gerven T., Braeken L. (2017). Chem. Eng. Process..

[cit303] Bremond N., Arora M., Ohl C.-D., Lohse D. (2006). Phys. Rev. Lett..

[cit304] Zhang L., Belova V., Wang H., Dong W., Möhwald H. (2014). Chem. Mater..

[cit305] Vasseghian Y., Dragoi E.-N., Almomani F., Le V. T. (2022). Chemosphere.

[cit306] Al-Hamadani Y. A., Jung C., Im J.-K., Boateng L. K., Flora J. R., Jang M., Heo J., Park C. M., Yoon Y. (2017). Chem. Eng. Sci..

[cit307] Han B., Köhler K., Jungnickel K., Mettin R., Lauterborn W., Vogel A. (2015). J. Fluid Mech..

